# Structural and developmental dynamics of Matrix associated regions in *Drosophila melanogaster* genome

**DOI:** 10.1186/s12864-022-08944-4

**Published:** 2022-10-25

**Authors:** Rahul Sureka, Akshay Kumar Avvaru, Divya Tej Sowpati, Rashmi Upadhyay Pathak, Rakesh Kumar Mishra

**Affiliations:** 1 CSIR-Centre for Cellular and Molecular Biology, Uppal Road, Hyderabad, 500007 India; 2 Present Address: EMBL, Rome, Italy; 3 Academy of Scientific and Innovative Research (AcSIR), Ghaziabad, India; 4 Tata Institute for Genetics and Society, Bangalore, India

**Keywords:** Nuclear matrix, Matrix attachment regions, Chromatin organization

## Abstract

**Background:**

Eukaryotic genome is compartmentalized into structural and functional domains. One of the concepts of higher order organization of chromatin posits that the DNA is organized in constrained loops that behave as independent functional domains. Nuclear Matrix (NuMat), a ribo-proteinaceous nucleoskeleton, provides the structural basis for this organization. DNA sequences located at base of the loops are known as the Matrix Attachment Regions (MARs). NuMat relates to multiple nuclear processes and is partly cell type specific in composition. It is a biochemically defined structure and several protocols have been used to isolate the NuMat where some of the steps have been critically evaluated. These sequences play an important role in genomic organization it is imperative to know their dynamics during development and differentiation.

**Results:**

Here we look into the dynamics of MARs when the preparation process is varied and during embryonic development of *D. melanogaster*. A subset of MARs termed as “Core-MARs” present abundantly in pericentromeric heterochromatin, are constant unalterable anchor points as they associate with NuMat through embryonic development and are independent of the isolation procedure. Euchromatic MARs are dynamic and reflect the transcriptomic profile of the cell. New MARs are generated by nuclear stabilization, and during development, mostly at paused RNA polymerase II promoters. Paused Pol II MARs depend on RNA transcripts for NuMat association.

**Conclusions:**

Our data reveals the role of MARs in functionally dynamic nucleus and contributes to the current understanding of nuclear architecture in genomic context.

**Supplementary Information:**

The online version contains supplementary material available at 10.1186/s12864-022-08944-4.

## Background

The eukaryotic genome needs to fit in a relatively small sized nucleus. To achieve the high level of compaction, the DNA is wrapped around nucleosomes, and the resulting 30 nm chromatin fiber is further organized into higher order organizations. The compaction however does not act as an impediment to nuclear functions. Instead, the packaging of the genome adds a layer of regulation on transcriptional output by limiting the access to genes in a dynamic and controlled manner. Genomic studies have revealed a hierarchically folded higher order chromatin organization in the interphase nucleus which consists of chromosome territories, genomic compartments and topologically associated domains. The folding of genomes into epigenomic compartments is now relatively well understood using chromatin conformation capture-based methods which uses proximity ligation of genomic loci in vivo to estimate contact frequencies [1]. However, the structural constraint that is required to sustain these higher order organization of genome is thought to be provided by Nuclear Matrix (NuMat) [2, 3]. Along with this, the NuMat is proposed to provide a structural platform for dynamic nuclear processes like transcription, replication and repair [4]. NuMat is a biochemically defined structure, mostly made of RNA and proteins. Chromatin loops are attached to the NuMat with the help of Matrix Attachment Regions (MARs) at the base of such loops [5, 6]. The definition of MARs as loop anchoring sequences may imply that MARs have a purely structural role. This is not true as experimental characterization from eukaryotic genomes clearly indicate that several functions can be attributed to MARs including transcriptional activation, initiation of DNA replication, insulation of domains and chromatin remodeling [7–9].

Analysis of MARs reveal several unique features. MARs generally contain AT-rich sequences and can become single-stranded under torsional stress giving flexibility to the loops. They possess sequences for curved and /or kinked DNA, replication origins (ORI), binding motifs for Topo I/II and other NuMat proteins. Polypurine and polypyrimidine stretches that are known to form triple-helical structures are also frequently found in MARs. Individual MAR may not contain each of these motifs, but MARs in general, are enriched with such sequence features. In spite of knowing these properties of MARs, the identities of MARs at whole genome level remain unexplored, and thus their role in gene regulation is not completely understood. With recent advances of NGS techniques a few attempts have been made to identify MARs at the genome-wide scale [10, 11]. However, none of the studies addressed the dynamics of MARs during development and differentiation in any model organism. Since the NuMat proteome is known to be dynamic during development [12, 13], it is of interest to see if MARs follow a similar trend.

The protocol to isolate NuMat subjects the nuclei to sequential extraction by non-ionic detergents, nucleases and low/high ionic strength buffer. The residual nuclear framework which resists extraction is the NuMat [14]. However, several distinct protocols have been used to prepare this fraction. It has been shown that the protein composition of NuMat can vary depending on the subtle changes in isolation procedure. Further, RNase A treatment causes the collapse of anastomosing network. In the present study, we have compared the MAR sequences prepared by varying the isolation protocol to understand the changes that happen at molecular level.

Our results demonstrate that stabilization of isolated nuclei by incubation for a short time at physiological temperature, facilitates the isolation of a better preserved NuMat. RNA is a critical component of the architecture as its removal causes general collapse of the structure. *Drosophila* genome harbors a set of constitutive MARs, that exist in the unstabilized nuclei and are sustained even after RNase A digestion. Majority of these ‘Core-MARs’ are also present at all developmental stages. Interestingly, ~ 60% of the Core-MARs lie in peri-centromeric heterochromatin. They are mostly repeat sequences of some sort, like LINE, LTR, satellite repeat or simple sequence repeat. Apart from these Core-MARs, stabilization generates several dynamic MARs majorly in euchromatic region of the genome. Most notable amongst the dynamic MARs are the sequences from paused Pol II sites in 5’-UTR regions of genes. These 5’-UTR MARs are dependent on nuclear RNA integrity. They are dynamic during development and correlate with the transcriptional profile of the developmental stage. Based on these observations we postulate that the Core-MARs in pericentromeric heterochromatin may have a constitutive role like keeping the chromocenter intact and facilitating the expression of house-keeping genes embedded in pericentromeric heterochromatin. In euchromatin, the Core-MARs partition the genome into large topological loop domains. The large loops are temporarily subdivided into smaller units by the virtue of association of functional sequences (like transcription units) with NuMat and these appear as dynamic MARs. In summary, our study adds to the understanding of role of MARs in dynamics of nuclear architecture.

## Results

### Effect of stabilization on NuMat

**Fig. 1:**
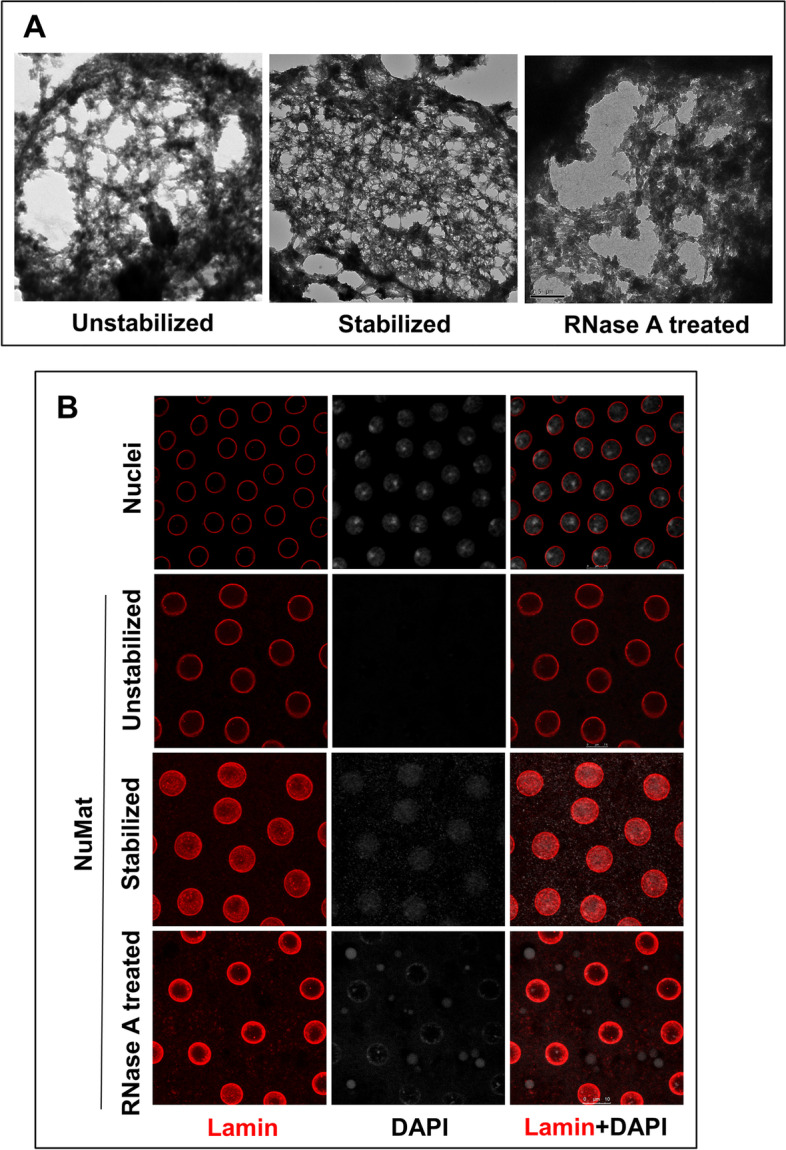
Effect of Stabilization and RNase A treatment on NuMat. **A** Resin less electron micrographs of unstabilized, stabilized and RNase A treated NuMat: The fine filaments seen in the stabilized NuMat are not seen in unstabilized NuMat. Instead, wide gaps with thicker fibers at the periphery are visible. The fine filaments seen in the stabilized NuMat collapse onto the peripheral lamina after RNase A treatment leaving wide gaps in the internal nuclear structure. **(B)** Confocal images of unstabilized, stabilized and RNase A treated NuMat: The nuclear panel shows presence of DNA as stained by DAPI. NuMat preparations are completely devoid of DNA. Internal lamin staining is visible in only in stabilized NuMat indicating it to be most intact

**Fig. 2:**
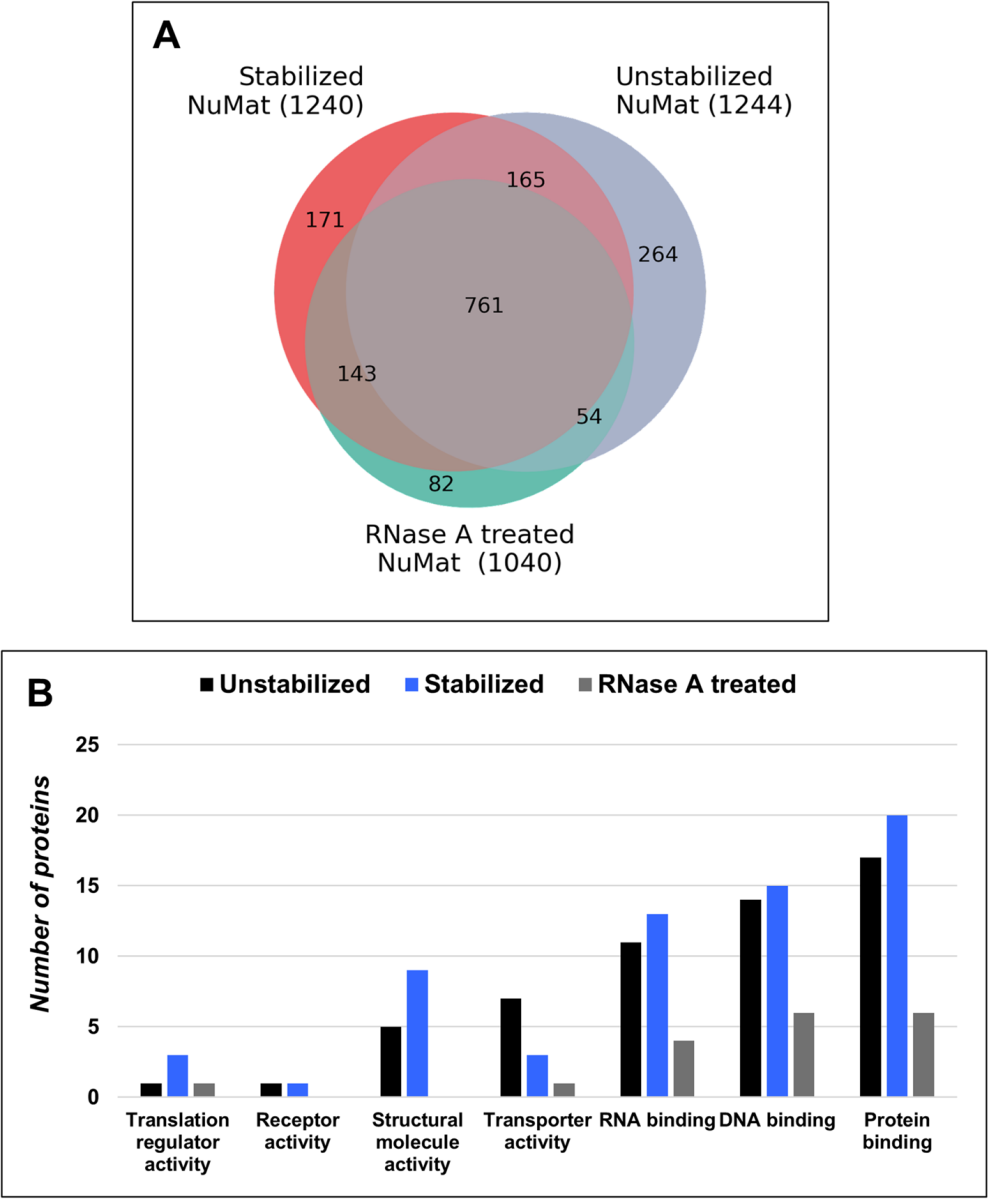
Protein composition of Unstabilized, Stabilized and RNase A treated NuMat. **A** Venn diagram to show the common and unique proteins between unstabilized, stabilized and RNase A treated NuMat. **B** Gene ontology terms enriched in the unique proteomes of unstabilized, stabilized and RNase A treated NuMat: Classification based on molecular function reveals that the unique proteins of both the unstabilized and stabilized NuMat preparations are involved in similar functions. Similar classification reveals that proteins involved in RNA binding are depleted from NuMat after RNase A treatment along with other DNA binding and structural proteins

In earlier studies, it has been observed that NuMat prepared with and without nuclear stabilization at 37 °C (unstabilized) had significant ultrastructural differences [15]. We validated these claims in *D. melanogaster*, by EM of resin-less sections of in situ NuMat prepared with 0–2 h embryos (Fig. 1A). The unstabilized NuMat shows gaps in the underlying structure and a reduced abundance of fine anastomosing filaments, otherwise seen in the NuMat prepared with stabilization. When observed under a confocal microscope using Lamin Dm0 antibody staining as a means of assessment, we see intra-nuclear Lamin Dm0 staining only after stabilization (Fig. 1B). Unstabilized NuMat appears as empty shell lined by the lamina without any internal lamin visible. This phenotype of lack of intra-nuclear Lamin Dm0 staining is seen in every nucleus from unstabilized or RNase A treated in situ NuMat preparation. To understand the molecular basis of these ultrastructural changes, we compared the biochemical composition of unstabilized and stabilized NuMat prepared from isolated embryonic nuclei. Quantification of nuclear DNA, RNA and proteins shows several folds increase in the amount of these macromolecules in stabilized NuMat [Supplementary Fig. 1]. To ascertain whether the increase in amounts of proteins is due to more types of protein molecules getting associated with NuMat or increase in quantity of a certain set of proteins, we carried out LC–MS/MS analysis. We identified 1240 proteins in NuMat prepared after stabilization and 1244 proteins in unstabilized NuMat. While 926 proteins were common, 318 and 314 proteins were unique to unstabilized and stabilized NuMat respectively (Fig. 2A) [Supplementary Table 1]. However, the unique proteins have comparable molecular functions (Fig. 2B). This indicates that functionally similar set of proteins are retained in unstabilized and stabilized NuMat, and stabilization results in an increase in their quantity.

**Fig. 3:**
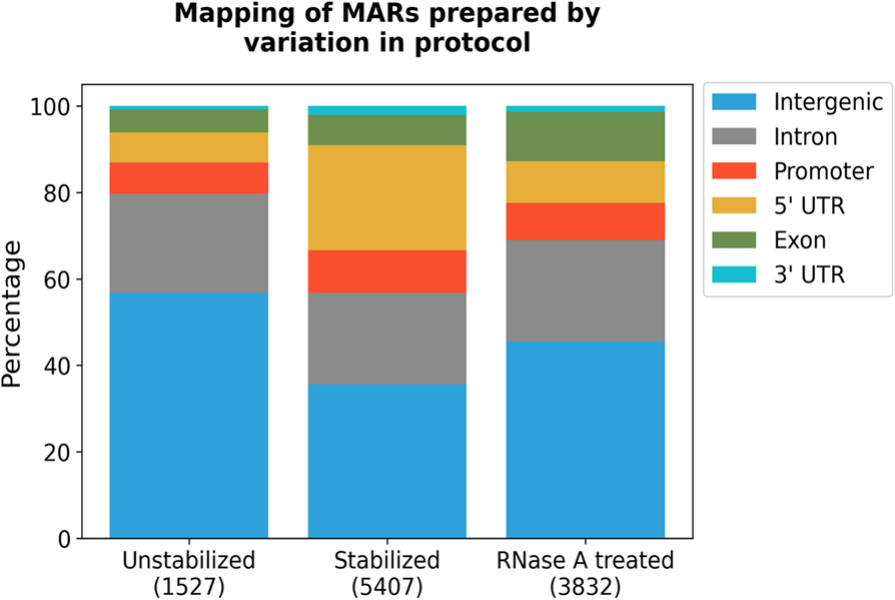
Genomic mapping of MARs prepared by protocol variation. The plot shows mapping of MARs in various categories with respect to gene position. The 5’-UTR MARs show significant increase after stabilization with a proportionate percentage decrease in intergenic MARs. RNase A digestion mostly effects the MARs in 5-UTR

Sequencing of MARs obtained from stabilized and unstabilized nuclei shows that the number of MARs increase ~ 3.5 folds upon stabilization [Supplementary Table 2]. Almost 62% of the unstabilized MARs are in the pericentromeric heterochromatin. After stabilization, the number of MARs increase predominantly in euchromatic region where they increase by ~ 6 folds as compared peri-centromeric heterochromatin where the number just doubles. The share of euchromatic MARs increases to 62% with concomitant decrease in heterochromatic MARs. In the context of known genes, MARs belonging to all the annotated categories increase after stabilization, however the most significant change is observed for 5’-UTR MARs, which increase more than ten-folds in number [Supplementary Table 3 and 4]. It is worth noting that after stabilization, many new MARs are generated within ~ 100 bp downstream of TSS, in the 5’-UTR. Thus, the share of MARs mapping to 5’-UTR increases from 7 to 24% with concomitant decrease in MARs mapping to intergenic region (Fig. 3). Earlier studies have shown that in ~ 10% of *Drosophila* genes, after recruitment, Pol II pauses at around ~ 50 bp downstream of TSS [16]. The striking feature of these paused Pol II genes is that they are developmentally controlled and poised for activation during subsequent stages of development. Comparing with the published data, we looked for the behavior of Pol II binding on the 5’-UTRs (1315 in number), that appear as MARs in stabilized NuMat. A sizeable fraction (70%—927 of 1315) of these 5’-UTRs are strongly disposed to Pol II pausing [Supplementary Table 5]. This observation indicates that stabilization of nuclei generates MARs at regions that are pre-disposed for Pol II pausing.

One interesting feature of MARs after stabilization is that most of the ~ 1300 MARs that are common between unstabilized and stabilized preparation, are a few folds enriched following stabilization [Supplementary Fig. 2]. This reiterates the point that stabilization consolidates pre-existing anchor points by strengthening the nuclear architecture.

### Functional significance of RNA in NuMat

In previous studies, the RNase A treated NuMat has been shown to be ultrastructurally similar to unstabilized NuMat [17]. To validate the observation, we prepared in situ NuMat from stabilized nuclei, with or without RNase A treatment. Comparison of EM pictures of resin-less sections of stabilized NuMat to RNase A treated NuMat preparations shows that RNase A treatment leads to collapse of NuMat fibers creating few but large gaps in the underlying filamentous meshwork (Fig. 1A). This picture is very similar to unstabilized NuMat indicating that the morphological integrity of NuMat observed after stabilization is largely dependent on RNA. Confocal imaging reiterates that upon RNase A treatment intra-nuclear architecture is disrupted as Lamin Dm0 staining is lost (Fig. 1B). To understand the molecular basis of these changes we compared the biochemical composition of untreated and RNase A treated NuMat. We quantitated the amount of nuclear DNA, RNA and proteins in untreated and RNase A treated NuMat to learn that while the amount of DNA and proteins did not change significantly, much of nuclear RNA was lost in RNase A treated NuMat [Supplementary Fig. 1]. Proteomic analysis identified 1040 proteins from RNase A treated NuMat and around 336 proteins are lost from NuMat following RNase A treatment (Fig. 2A) [Supplementary Table 1]. Gene ontology classification shows that many RNA/DNA binding proteins and structural proteins are lost from NuMat after RNase A treatment (Fig. 2B).

**Fig. 4:**
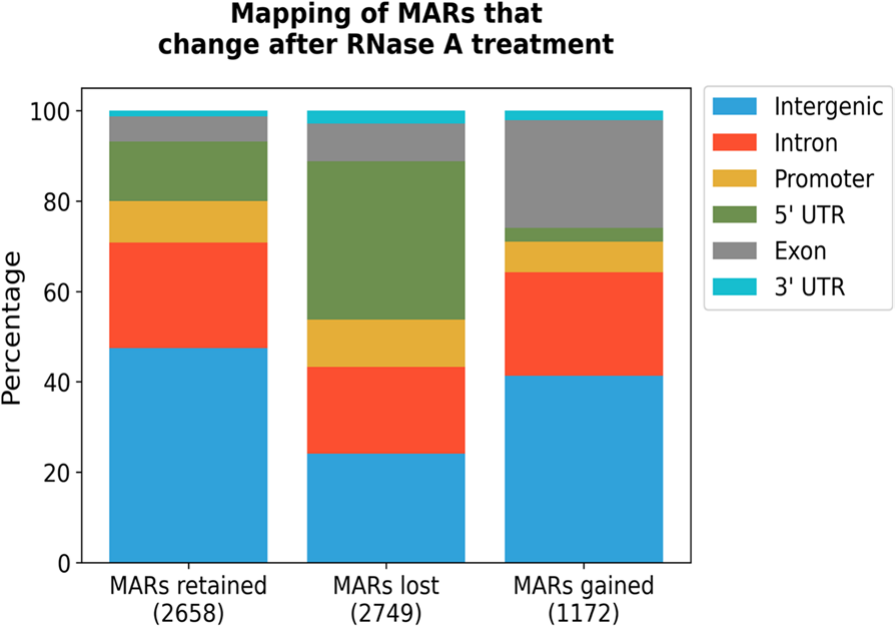
Analysis of MARs from RNase A treated NuMat. The plot shows mapping of MARs that change after RNase A treatment. The plot shows that significant proportion of MARs lost after RNase A treatment map to 5’-UTR and the ones gained map to exons and intergenic region

Sequencing of MARs from RNase A treated NuMat shows that 2749 MARs are lost upon RNase A digestion. Most of the lost MARs lie in euchromatin and annotate to 5’-UTR regions of genes (Fig. 3; Supplementary Tables 2 and 4). Interestingly, most of 5’-UTR MARs lost after RNase A digestion are the same ones that were gained as MARs following stabilization. This observation raises the possibility that the paused Pol II sites associate with NuMat in RNA dependent manner. We have tested this possibility as described in next section. However, along with 2749 MARs that are lost, a good number of MARs (1172 in number) are gained after RNase A digestion of NuMat (Fig. 4). These MARs comprise an intriguing category. In the protocol, RNase A digestion was done along with DNase I digestion in the nuclei. This was followed by salt and detergent extractions to prepare NuMat. As nuclear architecture suffers significant damage upon RNase A treatment, it appears counter intuitive that biologically relevant new attachment sites would be generated in such condition. However, the digested pieces of DNA would still be around and can get randomly associated with the degrading NuMat. We observe that ~ 60% of these newly generated MARs in RNase A digested NuMat lie in euchromatin. Annotation shows that close to ~ 60% of them associate with gene body while ~ 40% are intergenic (Fig. 4). However, we did not find any special sequence feature among these MARs nor do they harbor any specific motif or sequence repeat. They also do not show skewed AT or GC richness. On careful observation we could find some difference in the size of MARs lost and MARs gained after RNase A treatment, the former being smaller in size than the latter [Supplementary Fig. 3]. These observations suggest that digestion of nuclei with RNase A during NuMat preparation causes a collapse of nuclear architecture and apart from many MARs that are lost from 5’-UTR region, many genic DNA sequences get associated randomly with the degrading structure resulting in new MARs.

### Promoter MARs get associated with NuMat in an RNA dependent manner

**Fig. 5:**
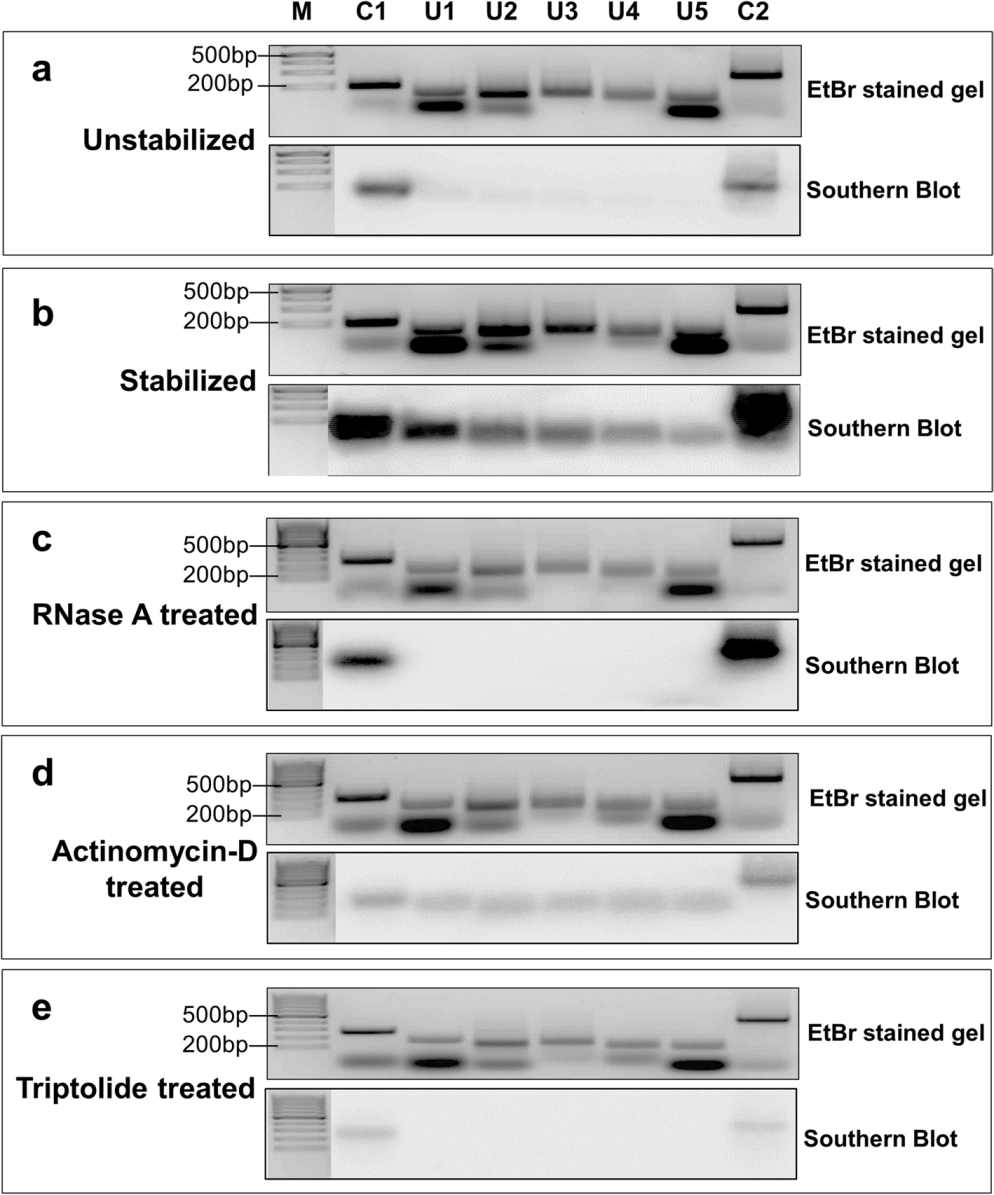
Validation of MARs and effect of transcription inhibitors on MARs. Southern blot validation for seven 5’-UTR MARs chosen on the basis of sequencing data. C1 and C2 are control MARs present in stabilized, unstabilized or RNase A treated NuMat. U1 to U5 are unique MARs present only in stabilized NuMat. The upper picture in each panel (a to e) shows the EtBr-stained gel profile of PCR amplified MAR sequences from genomic DNA. The lower picture shows the blot probed with ^32^P labelled MAR DNA obtained from NuMat isolated under condition as indicated. The C1 and C2 MARs are NuMat associated under all circumstances, whereas U1 to U5 MARs are gained after stabilization and lost after RNase A treatment. Their intensity reduces after actinomycin-D treatment and is completely lost after triptolide treatment. The gels and the blots have been cropped from top and bottom to fit into a single panel for easy comparison

The above results indicate that a significant proportion of MARs in 5’-UTR regions are associated with NuMat during the stabilization process. This association is RNA dependent as it is lost after RNase A treatment. To validate these results, we randomly selected 2 promoter MARs, represented by control 1 and control 2 (C1 and C2), that always remain associated with NuMat, irrespective of the isolation procedure. Thus, these sequences appear as MARs in unstabilized, stabilized (untreated) and RNase A treated NuMat. On the other hand, 5 promoter MARs represented by unique 1–5 (U1-U5), that associate with NuMat in RNA dependent manner during stabilization, were chosen. These promoter MARs are gained after stabilization and are lost upon RNase A digestion of NuMat. All of these sequences (C1-C2 and U1-U5) were PCR amplified, resolved on an agarose gel and transferred to nylon membrane. Southern hybridization was carried out with α^32^P-dATP labelled MAR-DNA prepared from unstabilized, stabilized and RNase A treated NuMat. We observe that in agreement with sequencing data, C1 and C2 appear as MARs in NuMat prepared by all the three methods, U1-U5 appear to be NuMat associated only in stabilized NuMat but not in unstabilized or RNase A treated NuMat (Fig. 5; Supplementary Fig. 5). To further test whether these associations are dependent on ongoing-transcription, we introduced two transcription inhibitors, actinomycin-D and triptolide. These inhibitors were introduced in the isolated nuclei along with the stabilization step, before NuMat was prepared from the nuclei. Actinomycin D and triptolide are transcription inhibitors with varying mechanism of action. Actinomycin D intercalates between the DNA and inhibits Pol II elongation. Triptolide completely arrests transcription initiation and leads to rapid degradation of Pol II [18]. We see that after actinomycin D treatment, association of MARs U1-U5 to NuMat is reduced. However, triptolide treatment abolishes the association completely and shows a pattern similar to unstabilized or RNase A treated NuMat (Fig. 5). Our results indicate that during stabilization of nuclei at 37 °C, transcription initiation is followed by elongation and stalling of Pol II. The nascent RNA generated aids in NuMat association of the locus. As actinomycin D inhibits elongation, it reduces NuMat association to some extent but does not abolish it. However, as triptolide inhibits transcription initiation itself, it wipes out NuMat association completely.

### MARs are developmentally dynamic

In order to understand the developmental dynamics of MARs in vivo, we used 0–2 h (early), 6–8 h (middle) and 14–16 h (late) stage developing *Drosophila* embryos. Isolated nuclei were stabilized and NuMat was prepared from each of these stages. MARs were isolated from the NuMat and sequenced using Illumina platform. Three biological replicates were used for each developmental stage which produced a total number of reads ranging from 44 – 66 million per sample. Out of these, 81–93% of the reads aligned to the dm6 genome build of the *D. melanogaster* genome [19]. Peaks that were common in all the three biological replicates were considered as MARs for each developmental stage. A total of 1228, 7284 and 5382 MARs were identified in the early, middle and late stages of development respectively [Supplementary Table 6]. MARs at each of these stages vary in size from 50-200 bp and occur at genomic distances ranging from < 1 kb to 150 kb with an average inter-MAR distance of 30 kb.

**Fig. 6:**
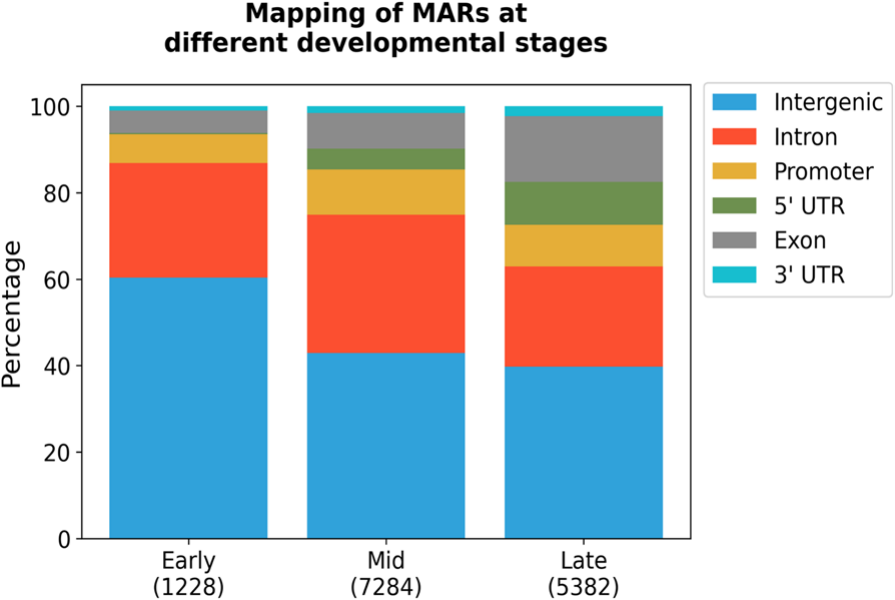
Mapping of MARs at different developmental stages. The plot shows the percentage of MARs in various annotated categories at different developmental stages. The 5’-UTR and exonic MARs increase in number through development. The intergenic MARs show a proportional decrease

**Fig. 7:**
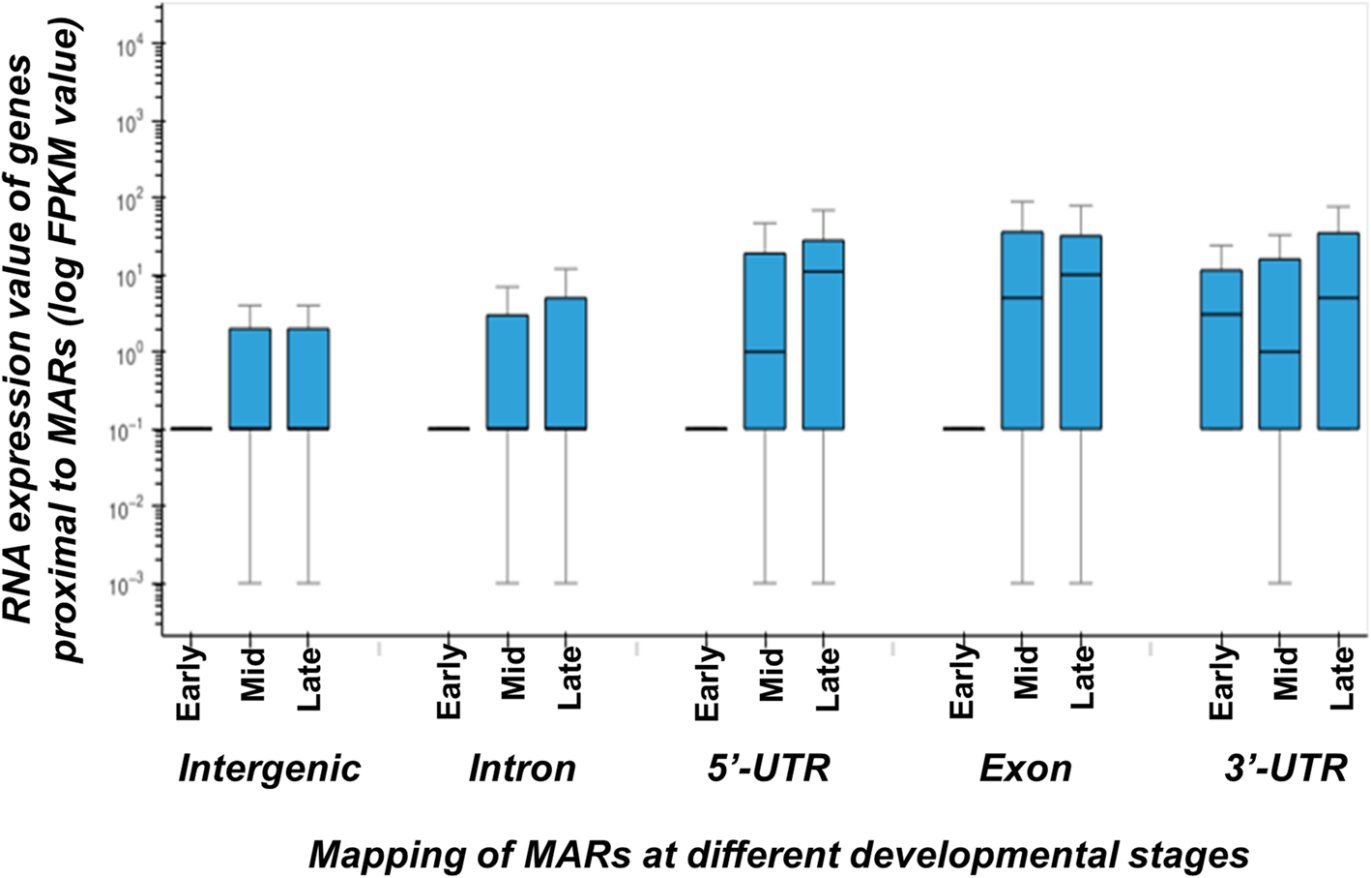
Genes with MARs in transcribed and protein coding regions are highly expressed**. Plot** to compare expression of genes having MARs in different regions at all three developmental stages. Genes are grouped into five categories depending on MAR association in either intergenic, intronic, 5’-UTR, exonic or 3’-UTR regions. Genes with MARs in transcribed regions of 5’-UTR, exon and 3’-UTR, have higher expression (FPKM value) as compared to those that have MARs in intergenic and intronic regions

Genomic location of these MARs is mapped with respect to gene bodies [Supplementary Table 7]. We find that as embryos develop, the number of MARs increase across the genome. The MARs occurring in 5’-UTR, exons and 3’-UTRs of genes, gradually increase in number during development. However, the number of MARs in intronic and intergenic regions increase significantly from early to middle stages and then decrease at later stage of development. Noticeably, in 0–2 h embryos where transcriptional activity is negligible, the number of unique genes which have a MAR associated at the 5’-UTR is only 3. Number of MARs at other genic regions is also low this early in development, and 60% of the MARs are found in intergenic regions (Fig. 6). At later developmental stages, MARs in genic regions increase with concomitant decrease in intergenic region. Interestingly, the number of MARs in 5’-UTR, 3’-UTR and exons keep increasing from mid to late developmental stages, whereas MARs in promoter, introns or intergenic regions, increase from early to middle stage and then decreases at later stage embryo. To understand the significance of this bimodal dynamics, we compared these results with the gene expression levels. RNA sequencing data for the three developmental stages was downloaded from the modENCODE database. The expression levels (FPKM value) of the genes with MARs in the 5’-UTR, exonic, intronic, 3’-UTR or intergenic region at different developmental stage was plotted (Fig. 7). We find that genes that associate with NuMat at the 5’-UTR, 3’-UTR and exons are highly expressed at that particular developmental stage, compared to genes which are associated with NuMat at intronic or intergenic regions. This observation indicates that MARs in UTRs and exons correlate with enhanced gene expression.

### Chromatin features of MARs

**Fig. 8:**
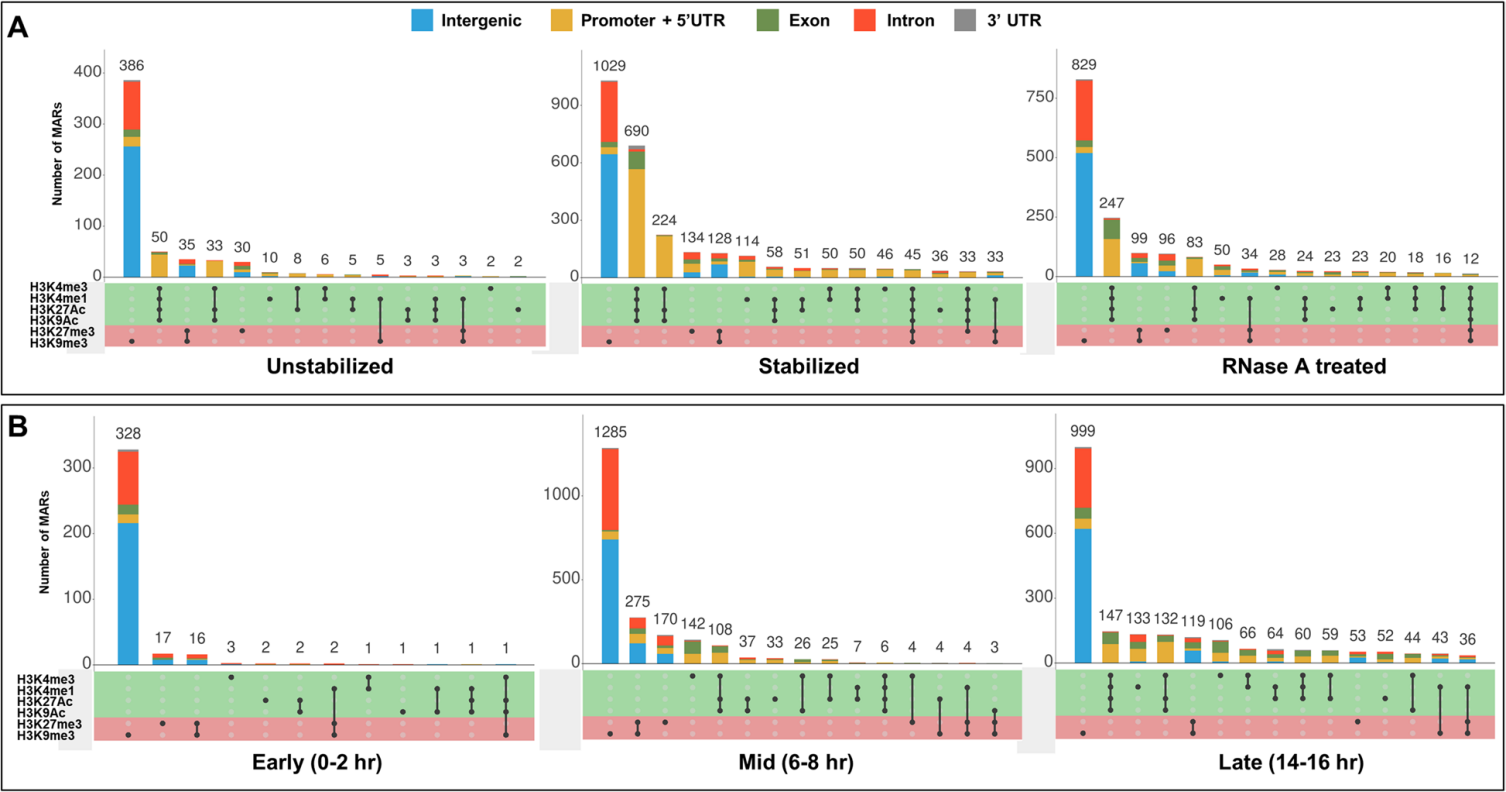
Epigenetic profile of MARs. Plot shows distribution of modified histones over MARs prepared by variation in protocol (**A**) and different developmental stages (**B**)

Histone modifications represent epigenetic signatures that relate to functional status of chromatin. To get an idea of functional attributes of MAR sequences, we looked into their chromatin features. Interestingly ~ 50 to 60% of MARs are nucleosome free as not even H3 was found to be present on these sequences [Supplementary Fig. 4]. We checked for the overlap of repressive chromatin marks (H3K9me3 and H3K27me3), active promoters marks (H3K4me3 and H3K9ac) and active enhancers marks (H3K4me1 and H3K27ac) with MAR sequences (Fig. 8). The repressive chromatin mark of histone modified at H3K9me3 is the most abundant mark and it is mostly present on intronic and intergenic MARs. Conforming to the present understanding, MARs in promoter and 5’-UTR regions, carry nucleosomes containing histones modified at H3K4me1, H3K4me3, H3K9ac and H3K27ac. Overall we can say that almost 50% of the MARs are nucleosome free stretch of DNA in close contact with nuclear architecture, although this needs to be validated by further experiments.

### Protein factors binding at MARs

**Fig. 9:**
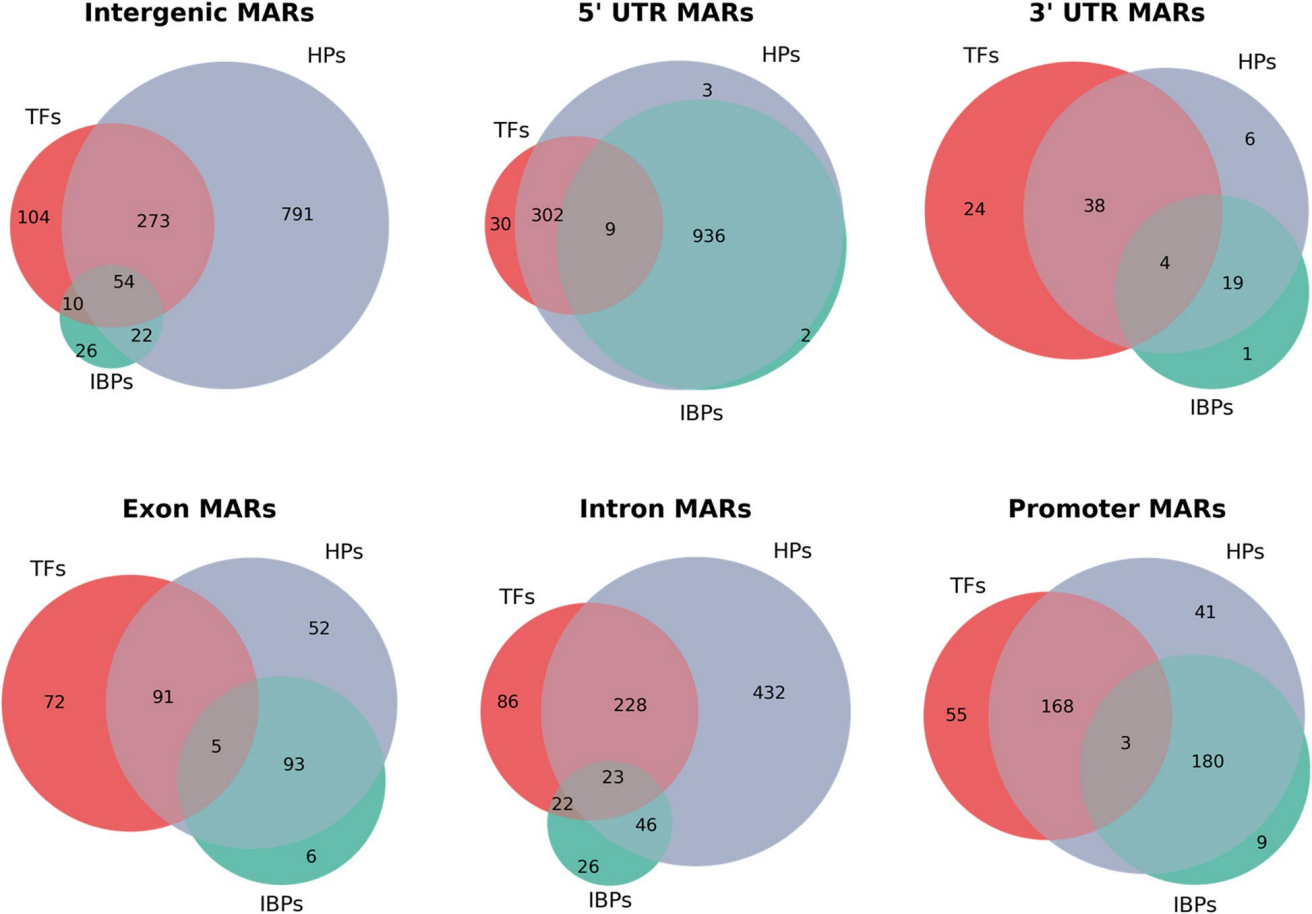
Protein factors binding at MARs. Venn diagrams to show types of proteins that bind to MARs of different categories. ChIP-Seq data for protein binding has been obtained from modENCODE. Transcription factors (TFs) – lola, Trithorax-like, fruitless, kruppel, knirps, caudal, hairy, homothorax, pangolin, paired, ultraspiracle, Polycomb-like, yorkie, scute, huckebein, Su(H), Hr46, Hr78, Eip74EF, Stat92E, MBD-R2. Insulator binding proteins (IBPs) – BEAF32, CP190, CTCF, Su(Hw), Mod(mdg)4, Zw5. Heterochromatin proteins (HPs) – HP1a, HP1b, HP1c, HP2, HP4, Su(var)3–7

As a *cis*-acting element, function of a MAR would depend on the *trans*-acting factors that binds to it. We looked into the types of protein factors that have binding sites that overlap with MAR sequences. We looked into the matching ChIP-sequencing data with 0–16 h embryo available at modENCODE. As presented in Fig. 9 and Supplementary Table 8, we find that heterochromatin proteins (HPs) are the largest category of proteins that bind to MARs of all annotated categories. MARs in the 5’-UTR are most decorated ones as most of them bind to some protein factor. A majority of them bind to transcription factors (TFs) along with HPs (936 of 1315) while a significant subset binds to insulator binding proteins (IBPs) along with HPs (302 of 1315). Intergenic MARs are occupied to a lesser extent with almost 1/4^th^ of them (486 of 1924) do not bind to any of the factors queried. More than 1/3^rd^ intergenic MARs are bound to only to HPs. Intronic MARs show a pattern of binding similar to intergenic MARs and promoter MARs show a pattern similar to 5’-UTR MARs (Fig. 9). This analysis indicates that most MARs function as *cis*-elements defined by the properties of protein factor binding to it. However, a subset that does not bind to any protein factor need to be studied further for special features.

### Core-MARs in D. melanogaster genome

**Fig. 10:**
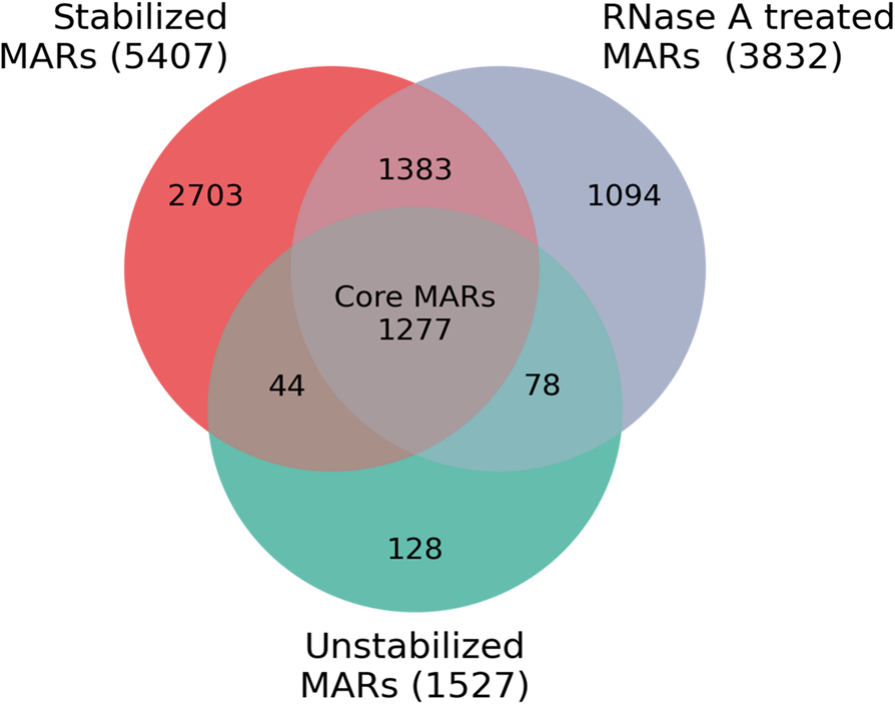
Venn diagram to compare MARs prepared by variation in protocol. Unstabilized NuMat has 1527 MARs that increase to 5407 upon stabilization. RNase A treatment reduces the number to 3832. The 1277 MARs present in all the conditions are termed as Core-MARs

We find that 1277 MARs are common between different methods used to isolate NuMat (Stabilized, Unstablized, RNase A treated) (Fig. 10). Of these 943 are present at all the three developmental stages examined. We call them as the Core-MARs in *D. melanogaster* genome [Supplementary Table 9]. The Core- MARs show several interesting features.

**Fig. 11:**
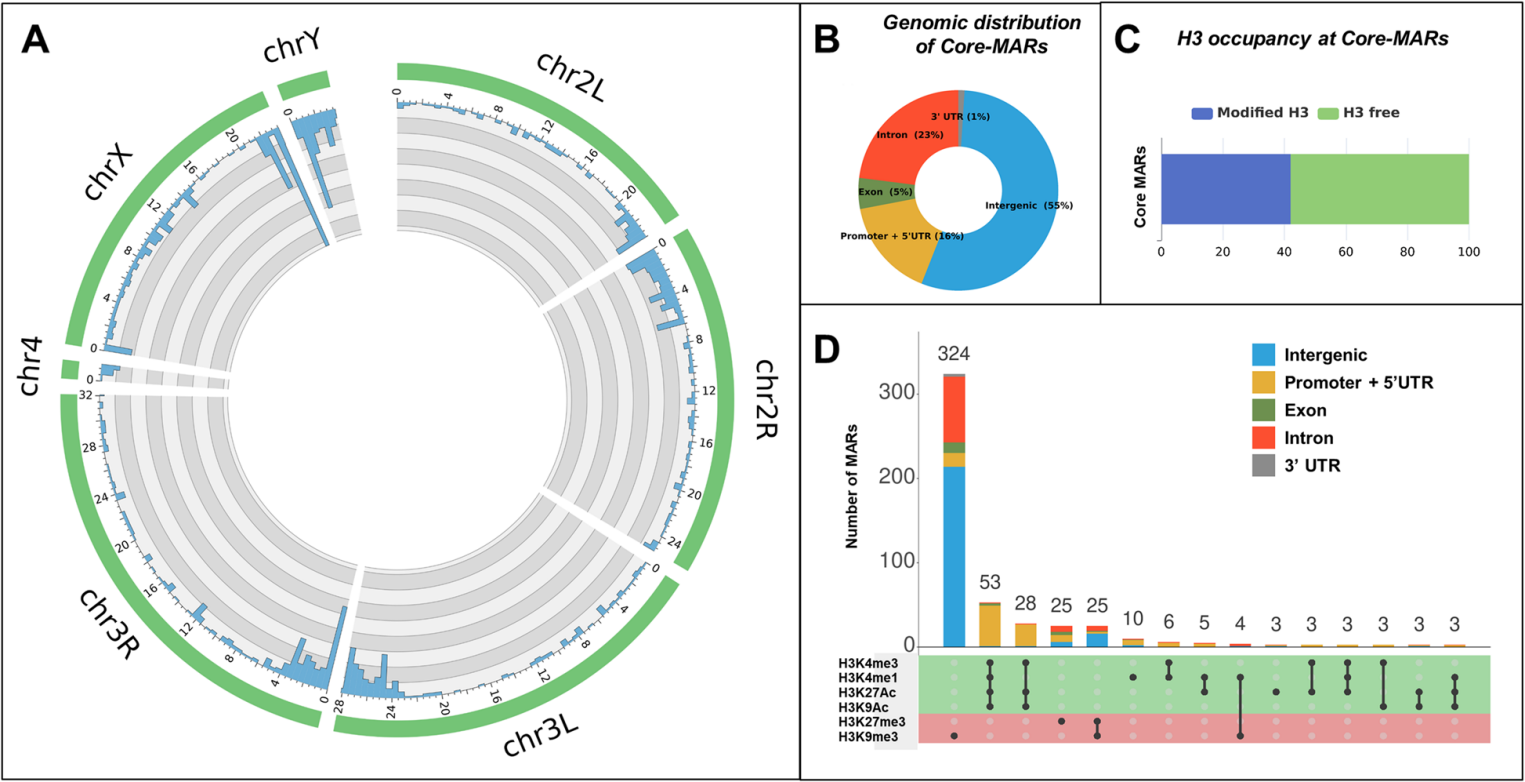
Core-MARs across the *D. melanogaster* genome: **A** Circos plot to show the density of Core-MARs on different chromosomes of *D. melanogaster*. Blue columns show enrichment of MARs in pericentromeric regions and Y chromosome. **B** Pie chart to show percentage of Core-MARs mapping to various genomic regions. **C** Plot to show that 60% of Core-MARs are histone free. **D** Epigenetic profile of Core-MARs to show that repressive histone mark of H3K9me3 is predominantly present on intergenic and intronic MARs

- Mapping of Core-MARs shows that they are enriched in intergenic and intronic regions (55 + 23 = 78%) (Fig. 11B).
- A circos plot in Fig. 11A shows the density of Core-MARs (represented by blue columns) on different chromosomes of *D. melanogaster*. We find an enrichment of Core-MARs on chromosomes X and Y and peri-centromeric regions of the autosomal chromosomes. Accordingly, we find that 797 of the Core-MARs lie in pericentromeric heterochromatin and 480 in euchromatin.
- Almost 60% of the Core-MARs are histone free and in line with the general MARs they too have H3K9me3 as the major modified histone mark overlapping with intergenic and intronic MARs (Fig. 11C and D).
- An overwhelming majority (83%) of Core-MARs are sequences from repetitive region of the genome (Table 1). The intergenic and intronic Core-MARs are enriched with retroelements LINEs and LTRs followed by satellite DNA. Whereas the 5’-UTR and exonic Core-MARs are low in repeats and mostly have SSRs as repetitive component.
- Around 40–60% of the Core-MARs (with the exception of 5’-UTR and 3’-UTR Core-MARs) associate with pseudogenes or non protein-coding genes (Table 2).

**Table 1:** Association of Core-MARs with repeat elements

Types of Core-MARs	Number	Associated with repeats		Repeat Type					
		**Number**	**%**	**LINE**	**LTR**	**DNA**	**SSR**	**Sat**	**rDNA**
**Intergenic**	**703**	**665**	**95**	**220**	**256**	**35**	**22**	**122**	**10**
**Promoter**	**112**	**69**	**62**	**6**	**13**	**9**	**27**	**7**	**7**
**5’UTR**	**95**	**16**	**17**	**0**	**0**	**0**	**16**	**0**	**0**
**Exon**	**59**	**19**	**32**	**0**	**1**	**2**	**10**	**1**	**5**
**Intron**	**297**	**280**	**94**	**75**	**117**	**43**	**16**	**29**	**0**
**3’UTR**	**11**	**8**	**73**	**1**	**4**	**0**	**2**	**1**	**0**
**Total**	**1277**	**1057**	**83**	**302**	**391**	**89**	**93**	**160**	**22**

**Based on comparison of MAR sequences with *Drosophila* reference genome build dm6**

**Table 2:** Association of Core-MARs with non-coding RNA genes

Types of Core-MARs	Number	Associated with protein coding gene		Associated with non-coding RNA genes		Total Core-MARs associated with non-coding RNA genes	
		**Number**	**%**	**miRNA** **rRNA** **tRNA** **snRNA** **lncRNA**	**Pseudogenes**	**Number**	**%**
**Intergenic**	**703**	**397**	**56**	**282**	**24**	**306**	**44**
**Promoter**	**112**	**67**	**60**	**33**	**12**	**45**	**40**
**5’UTR**	**95**	**95**	**100**	**0**	**0**	**0**	**0**
**Exon**	**59**	**24**	**41**	**25**	**10**	**35**	**59**
**Intron**	**297**	**181**	**61**	**75**	**41**	**116**	**39**
**3’UTR**	**11**	**9**	**82**	**2**	**0**	**2**	**18**
**Total**	**1277**	**773**	**60**	**417**	**87**	**504**	**40**

**Based on comparison of MAR sequences with *Drosophila* reference genome build dm6**

## Discussion

Our ability to understand how genomic DNA is spatially organized into eukaryotic cells has dramatically increased with the development of robust proteomics and deep sequencing techniques. These technologies in conjunction with techniques like chromatin conformation capture (3C), ChIP sequencing, high resolution imaging and genome modeling have started to address the important question of how higher order structure of DNA regulates cell-specific expression of genes. However, NuMat attachment sites of chromatin have seldom been applied in this context. In the present study, we have sequenced the MARs of *D. melanogaster* at developmental stages to understand the role of these sequences in genome packaging and regulation.

Before investigating the dynamics of MARs during development and differentiation, we re-visited the NuMat isolation protocols. Initial studies have suggested that the nuclear protein matrix is mostly made up of lamins. Subsequently, the protein composition has been found to be more complex with lamins being the most prominent and reproducibly associated protein [20, 21]. It is observed that protein composition depends on subtle variations in the isolation protocol. The protein composition is notably enriched when NuMat is prepared in the presence of oxidative agents or divalent cations [22, 23]. Similar effect is seen if the NuMat is prepared with inclusion of a heat stabilization step where the isolated nuclei is briefly exposed to physiologic temperature of 37 °C prior to salt extraction [15, 24]. EM images show that stabilization is necessary for detection of fibro-granular network inside the nuclei. The stabilization step is particularly questioned, as it has been shown to render a set of nuclear proteins insoluble by creating disulfide cross-links [22, 25]. It is important to mention here that nuclear proteins are not cross-linked by disulfide bonds in vivo. Such cross-links appear spontaneously during isolation of nuclei and NuMat, and thus if alkylating agents are used to inhibit the formation of disulfide bonds, the NuMat prepared subsequently is barely recognizable [22, 26]. These observations lead to a critical question that whether the isolation process stabilizes a pre-existing structure or causes changes in the nuclear organization to create such structure. We addressed this in our present study by comparing the NuMat proteome and MAR sequences isolated from unstabilized and stabilized nuclei. Our results demonstrate that in agreement with the published literature, intricate and dense fibro-granular meshwork can be visualized after stabilization. Protein content of NuMat isolated with or without stabilization remain qualitatively similar, although there is a significant quantitative increase after stabilization. Therefore, upon stabilization, more amounts of same proteins are retained in the NuMat, increasing the amounts of nuclear proteins retained in NuMat from 4 to 10%. MARs too show a similar trend as the unstabilized MARs are further enriched upon stabilization. These observations strengthen the possibility that internal NuMat exists in live cells and interventions like exposure to 37 °C stabilizes it and facilitate its isolation.

Further, it has been shown that upon RNase A treatment, the meshwork of fine filaments populating the NuMat, collapses and aggregates onto the nucleolus and peripheral lamina. In order to understand the molecular details of this morphological effect and to determine the structure–function relationship of NuMat RNA, we have compared the NuMat proteome of RNase A treated versus untreated nuclei. Imaging analysis shows that large gaps appear in the meshwork on RNase A digestion. Many of the structural proteins like zipper (non-muscle myosin heavy chain), beta-spectrin, beta-tubulin, etc., are lost from the NuMat. Along with them, several transcription factors and RNA binding proteins are also lost upon removal of RNA from the structure. This confirms that the dynamic association of various proteins in the NuMat is managed by NuMat RNA, which plays an integral part in holding the functional matrix together.

Sequencing of MARs isolated from unstabilized, stabilized and RNase A treated NuMat provides further insight into the molecular dynamics of nuclear architecture. Apart from a set of sequences, that we call as Core-MARs here, most of the MARs are dynamic. The MARs occurring in promoter region and 5’-UTR of genes are the most dynamic of them all. They get associated with the NuMat during the stabilization of nuclei at 37 °C, in an RNA dependent manner, since the same promoter and 5’-UTR MARs that are gained by stabilization, are lost after RNase A treatment of NuMat. The association of the MARs during stabilization possibly happens due to initiation of transcriptional activity and pausing of Pol II. Stalled Pol II has been shown to accumulate just downstream of the promoters in several previous studies and such promoters have already been found enriched in NuMat [10, 27, 28]. Pol II pauses proximal to the promoter, where it waits for proper cellular cues to proceed for elongation. This mechanism provides a number of advantages in terms of rapid and synchronous activation of genes. Interestingly, some promoters show a strong tendency for Pol II pausing and this property is tightly linked to DNA sequence of the promoter [27, 29]. We also observe that RNase A digestion not only detaches the promoter and 5’-UTR MARs from protein matrix, it also disrupts the architecture significantly. Our study sheds light on the critical debate about the mechanism by which stabilization makes the NuMat filaments more robust to EM observation. Our results suggest that the transcriptional activity and stalling of Pol II makes the NuMat fibrils more stable, and that this stability is dependent on, and is mediated by the transcribed RNA.

We next explored the dynamics of MARs as development proceeds in *D. melanogaster* embryos. Earlier studies from our group has shown that 65% of NuMat proteome in fruitflies is dynamic during development [30]. Here we find that just like the protein composition of NuMat, MARs can also be either stable or dynamic. Stable MARs do not change between cell types as the embryo develops, which we have classified as Core-MARs. Dynamic MARs are the ones which change during development and are cell type dependent. We observe that 1277 MARs are stably associated with NuMat in 0–16 h old embryos of which 943 MARs are maintained at all the three stages of development examined. They are distributed throughout the genome, with enrichment at pericentromeric region and on X chromosome. Published studies from our lab has shown that in pericentromeric heterochromatin, MARs correlate well with TADs and possibly play an instrumental role in expression of genes embedded in heterochromatin [31, 32]. Also in *Drosophila*, centromeres of chromosomes are clustered into discrete nuclear structure known as chromocenter. Chromocenter ensures encapsulation of all chromosomes into a single nucleus and its disruption leads to micronuclei formation ultimately resulting in cell death [33]. It is possible that Core-MARs play a basic role in maintenance of the nuclear architecture by helping in bundling and anchoring of chromocenter. More than half of the Core-MARs are in intergenic regions of the genome suggesting that these regions help in stably anchoring the genome to the underlying NuMat. Increased density of Core-MARs on X chromosome might be linked to the higher activity of genes for dosage compensation. The next abundant category is intronic MARs, which account for almost 1/4^th^ of Core-MARs. About 22% of the Core-MARs lie in promoter region, 5’-UTR, 3’-UTR and exons of genes. Here they show an interesting enrichment at pseudogenes and non-protein coding genes. Most of these pseudogenes are from rRNA cluster on X chromosome and suppressor of stellate cluster on Y chromosome. Pseudogenes are fossil copies of parent genes, and are traditionally thought to be nonfunctional elements. Recently, however, some of them have been shown to be transcribed and are implicated in regulation of protein-coding genes. A comparative genomic study demonstrates that pseudogenes are highly linage specific and reflect genome history [34]. NuMat association of such lineage specific elements as constitutive Core-MARs, proposes an interesting lineage specific feature of MAR elements. However, the significance of pseudogenes as Core-MARs, remains to be explored further.

Among the dynamic MARs, the most dramatic changes are observed in the MARs associated with the promoters and 5’-UTR of genes. The genes with MARs in their promoters and 5’-UTR regions are amongst the most highly expressed genes and are pre-disposed to Pol II pausing at promoter. Accordingly, only three MARs show up in the 5’-UTR regions in early embryos where transcription is negligible. It is known for a long time that the positioning of a gene at the NuMat increases transcription. For example, in “halo” preparations where loops of DNA stripped of histones remain attached to the NuMat, active genes localize to the bases of loops while inactive genes are peripheral [35]. Our data too indicates that attachment to NuMat at promoter, UTRs and exonic region is, more often than not, involved in enhanced gene expression and NuMat provides the necessary platform for active transcription as well as anchors genes poised for transcription.

A curious observation from our study was that the total number of MARs increased from 0–2 h embryos to 6–8 h embryos but decreased in 14–16 h embryos. This is intriguing because, as the number of cell types increase with development, number of MARs are expected to increase at later stages. One possible reason might be that for many of the dynamic MARs, originating from the increased number of cell types at later stages, may have increased the signal to noise ratio. In such a case, the below thresh-hold reads coming from a small number of cells, may have been removed from the dataset during analysis. The other possibility can be that MARs represent more of active regulatory elements like enhancers, repressors and insulators, that are in action at middle stages of development when the cell lineages are being decided. The regulatory activity reduces at later stages when cell fates are already assigned with concomitant decrease in number of MARs.

Epigenetic signature can be used to predict regulatory elements de novo. *Cis*-regulatory elements such as promoters, enhancers and insulators can be defined not only by the DNA sequence motifs but also by the patterns of bound proteins and histone modifications [36]. Going by the logic that function of MARs as *cis*-regulatory elements must be relative to the *trans*-acting factor that binds to it, we looked into the types of protein factors that have overlapping binding sites with MARs. Our analysis indicates that a significant proportion of MARs overlap with regulatory elements. Several MARs overlap with transcription factor binding sites, underlining the involvement of these elements in facilitating transcription as promoters and enhancers. Many promoter and 5’-UTR MARs overlap with binding sites of architecturally important and insulator binding proteins like CP190, CTCF, BEAF 32, GAF, Su(Hw). This is not surprising as these factors have been mapped by ChIP-Seq to frequently associate with TSS [37]. The HP1 family of proteins are found to bind mostly in intergenic region. However, a significant number of HP1 family of proteins binds with promoter MARs. This is also not surprising as a recent paper examined the genome-wide binding profile of HP1 family of proteins to find that in euchromatin, these proteins target genes that are highly expressed and exhibit Pol II pausing at TSS [38]. The clustered binding of many of these proteins at paused Pol II promoter MARs consolidates the idea that such promoters can also act as insulators [39]. A good number of intergenic and intronic MARs do not overlap with the binding of any of the protein factors examined. This maybe because ChIP data for known NuMat proteins (like NuMA, HnRNP-U/SAF-A, Lamin etc.) is not available in *Drosophila*.

Epigenetic profile of MARs shows an enrichment of H3K4me3 along with H3K9ac and H3K27ac, a pattern that signifies active promoters, over promoter and 5’-UTR MARs. Intronic and intergenic MARs mostly have repressive chromatin mark of H3K9me3. However, only ~ 30% of the MARs in intronic/intergenic MARs show such an epigenetic profile. A majority of MARs appear to be devoid of histones and can be categorized as nucleosome free regions. Summing up the protein binding and epigenetic profiles, we find that around ~ 35% of the intronic/intergenic MARs neither carry epigenetic profile conducive of enhancers, insulators nor do they have repressive chromatin marks. These MARs appear to be devoid of nucleosomes and probably associate with NuMat, purely based on their DNA sequence feature. This property of MARs needs to be analyzed further in detail.

## Conclusions

In conclusion, our data, supports the proposition that internal NuMat is an in vivo structure which needs stabilization for efficient isolation. RNA plays an integral role in organizing the functional NuMat and is responsible for tethering a subset of MARs to the architecture. Mapping of MARs from *D. melanogaster* genome during various stages of development reveals a strong link between these elements and transcriptional status. We show that with increasing developmental complexity the number of MARs increase, however there are Core-MARs remain stable throughout development. Epigenetic features indicate that MARs cohabit regulatory elements, like promoters, enhancers and insulators, although almost a quarter of them do not overlap with these classes of regulatory elements. 5’-UTR MARs are highly dynamic and RNA dependent for their association with the substructure. They are induced during the stabilization procedure, by the ongoing transcriptional activity and pausing of Pol II. In essence, Core-MARs are specific and static, and these sequences come mostly from the repetitive part of the genome. They are enriched in pericentromeric heterochromatin to play a constitutive role in nuclear architecture. Partitioning of the euchromatic genome into large topological loops by Core-MARs is subdivided into smaller units due to temporary association of transcribing units and their regulatory elements. These functional MARs are dynamic and cell type specific. Our study at genomic scale enhances the current understanding of structural basis for dynamic organization of the nuclear functions.

## Methods

### Preparation of NuMat and MAR DNA

Embryos (0–2, 6–8, 14–16 or 0–16 h of development) were collected from *Drosophila melanogaster* (Canton-S strain), and NuMat was prepared according to published protocol [10]. The following variations were included in the protocol 1) For unstabilized NuMat, the stabilization of nuclei at 37 °C for 20 min was excluded. 2) For RNase A treated NuMat, RNase A (2 µg/ml) was included along with DNase I during digestion step. 3) Actinomycin D or triptolide (1 µg/ml and 1 µM respectively) were included in the buffer during the stabilization step, in the respective NuMat preparations.

To isolate MAR DNA, isolated NuMat was treated with RNase A (10 µg/ml) at 37 °C for 30 min. This was followed by Proteinase K (100 µg/ml) digestion at 55 °C for 1 h. DNA was recovered by phenol:chloroform extraction and ethanol precipitation. The recovered DNA was further estimated by Qubit and processed for sequencing.

To compare DNA, RNA and protein retained in NuMat with reference to Nuclei, we isolated DNA, RNA and protein from NuMat/Nuclei and quantitated them. For DNA isolation NuMat/Nuclei samples were suspended in 0.5% SDS and treated with RNase A (50 µg/ml at 37 °C for 30 min). To this Proteinase K was added to a final concentration of 100 µg/ml and digestion was carried out at 55 °C for 2 h. DNA was precipitated after phenol:chloroform:isoamyl extraction. DNA was estimated using Qubit hs-DNA kit following manufacturer’s instruction. For RNA isolation NuMat/Nuclei samples were suspended in TRIzol and RNA was extracted using standard TRIzol protocol. The amount of RNA was also estimated using Qubit RNA kit following manufacturer’s instruction. For proteins, NuMat/Nuclei samples were dissolved in Laemmli’s buffer and heated at 95 °C for 5 min. Protein estimation was done using Pierce 660 nm protein assay reagent after addition of ionic detergent compatibility reagent and following manufacturer’s instruction.

### In-gel digestion and protein identification by LC–MS/MS

For tryptic digestion, 2-4 µg of protein were resolved on 12% SDS-PAGE. Gel slices were cut and dehydrated with sequential increase to 100% ACN. The gel slices were rehydrated with 100 µl-200 µl of Trypsin solution (10 µg/ml, Promega) in 25 mM ammonium bicarbonate. Digestion was carried out at 37 °C overnight and peptides were eluted with 50% ACN and 5% TFA, vacuum dried and stored at -70 °C till loaded on to the mass spectrometer.

The tryptic peptides were analyzed using standard columns with default settings on a 140 min gradient. The Proxeon LC system was directly connected with Thermo fisher scientific LTQ Orbitrap Velos instrument using Proxeon nanoelectrospray source. The nano source was operated at 2.2 kV and the ion transfer tube at 200 °C without sheath gas. The mass spectrometer was programmed to acquire in a data-dependent mode. The scans were acquired with resolution 60,000 at m/z 400 in Orbitrap mass analyzer with lock mass option enabled for the 445.120024 ion. The 25 most intense peaks containing double or higher charge state were selected for sequencing and fragmentation in the ion trap by using collision induced dissociation with a normalized collision energy of 40%, activation q = 0.25, activation time of 10 ms and one micro scan. Dynamic exclusion was activated for all sequencing events to minimize repeated sequencing. Peaks selected for fragmentation more than once within 30 s were excluded from selection for next 90 s, and the maximum number of the excluded peak was 500.

The raw spectra obtained were processed with Andromeda search engine (MaxQuant software version 1.1.0.39). Search was performed against *D. melanogaster* protein database UniProtKB *D. melanogaster database*. Trypsin/P was specified as the cleavage enzyme and up to two missed cleavages were allowed. The initial precursor mass tolerance was set at 10 ppm, and fragment mass deviation was set at 0.25 Da. The search included cysteine carbamidomethylation as fixed and oxidation of methionine as variable modification. The identification has been done keeping 1% false discovery rate at the peptide and protein level. Proteins identified with multiple peptides as well as one unique peptide with high confidence were listed and analyzed further.

### Next-generation sequencing of MARs

MAR DNA is already in the range of 50–100 bp, and very similar to ChIP DNA in size. Sequencing libraries were prepared using Illumina TruSeq ChIP sample preparation kit according to manufacturer’s instructions. Libraries were sequenced by single-end sequencing with 75 bp read length on the Illumina 2500 platform.

### Data processing and analysis

The quality of the raw sequencing data was checked using FastQC. The adapter and over-represented sequences were trimmed using Trimgalore (http://www.bioinformatics.babraham. ac.uk/projects/fastqc/). The reads were then aligned to the *D. melanogaster* reference genome build dm6 using Bowtie2 [40]. Blacklisted regions have anomalous unstructured, high signal/read counts in NGS experiments independent of the cell line and type of experiment. Hence, reads mapping to these regions were filtered. Also, reads which map to random scaffolds in the genome were filtered out, leaving reads mapping only to chromosomes 2L, 2R, 3L, 3R, 4, X, and Y. The list of blacklisted regions available for dm3 was downloaded (http://mitra.stanford.edu/kundaje/ akundaje/release/blacklists/dm3-D.melanogaster/) and lifted over to dm6 build using the Liftover (https://genome.ucsc.edu/cgi-bin/hgLiftOver) tool from the UCSC genome tools. Prior to peak calling reads below the mapping quality of 5 were filtered out.

Peaks were called on these filtered aligned reads using MACS2 on broad peak mode [41]. The minimum length of the peak is set to 50nt and the mfold range of the peak call model is set to 3–100. For each sample peaks of biological replicates were pooled. Peaks overlapping among all the biological replicates for considered as MARs of each condition. The genomic annotation was done using R package, from bioconductor library, ChIPseeker [42]. MARs for each condition were overlapped with ENCODE datasets (Supplementary Table 10) using BEDTOOLS intersect [43].

### Electron microscopy of NuMat samples

The NuMat pellet was washed twice with 0.1 M sodium cacodylate (EMS catalog no. 11654) at 4 °C. The samples were then fixed with 2% glutaraldehyde (EM grade) in 0.1 M sodium cacodylate for 30 min at 4 °C and washed with 0.1 M sodium cacodylate to remove the fixative. The samples were further post-fixed with 1% OsO_4_ in sodium cacodylate for 5 min, washed twice and then dehydrated using a gradient of increasing ethanol concentrations from 30 to 100% diluted in 0.1 M sodium cacodylate. The samples in 100% ethanol and were either embedded in resin or Diethylene glycol distearate (DGD).

For embedment free electron microscopy (EM), after dehydration, the samples were resuspended and incubated in 1:2 butanol:ethanol at RT for 15 min. This step was repeated with 2:1 butanol:ethanol and finally 100% butanol. The samples were embedded in DGD by gradually transitioning the samples from butanol to DGD with increasing concentrations of DGD and incubating for 15 min at 60 °C at each step. Finally, the samples were incubated at 60 °C with 100% DGD for 1 h. The blocks were allowed to cool, trimmed and sectioned using an ultramicrotome with a glass knife angle of 10°. The sections were picked on poly-L-lysine coated copper grids and allowed to dry. The DGD from the sections was removed by incubating the grids in butanol for 24 h and then dried using CO_2_ critical point drying. These were then imaged using a JEOL transmission electron microscope at 120 kV.

### Confocal microscopy of *in situ* NuMat samples

Drosophila embryos (0–2 h old) were used for in situ NuMat preparation as described earlier [44, 45]. An in-house developed anti-lamin Dm0 antibody, raised in guinea pig against the whole fly protein expressed in bacteria, was used at a dilution of 1:500 in PBT followed by Cy3 labeled secondary antibody. Embryos were then washed and mounted in Vectasheild with DAPI. Confocal laser scanning was carried out on a Zeiss LSM510 META (Carl Zeiss Inc) with excitation at 543 nm at a pinhole of 1 AU. Scanning was done in multi-track mode. The emission of Cy3 was acquired using 565–615 BP filter. Optical sections were taken at 0.35 um intervals. Individual optical sections were projected to give Zeiss LSM software version 3.2 SP2.

### *In vivo *MAR assay

To validate the sequences identified by sequencing as MARs, we performed in vivo MAR assay as described in [10]. Briefly, two promoter MARs (C1 and C2), that always remain associated with NuMat, irrespective of the isolation procedure and five promoter MARs (U1-U5), that associate with NuMat in RNA dependent manner during stabilization, were chosen. These sequences were PCR amplified from *Drosophila* genomic DNA using primers listed in Supplementary Table 11. The amplified fragments were resolved on 2% TAE agarose gel and transferred to nylon membrane using capillary transfer method. MAR DNA isolated from NuMat prepared with various treatments (stabilized, unstabilized, RNase A treated, actinomycin D treated, triptolide treated) were labeled with ^32^P-dATP by random primer labelling method. Southern hybridization was carried out for 16 h at 55 °C. The blots were washed stringently and imaged on a Phosphor-imaging screen. The PCR amplified fragments hybridize to corresponding ^32^P-labelled fragments (if available) in the MAR DNA preparation and show signal.

## Supplementary Information

**Additional file 1: Supplementary Figure 1. **Molecular composition of NuMat after stabilization and RNase A treatment. Amount of nuclear DNA, RNA and proteins retained in unstabilized, stabilized and RNase A treated NuMat: The amount of nuclear DNA, RNA and proteins retained in unstablized NuMat is significantly less than stabilized NuMat. The amounts of nuclear proteins retained in NuMat does not change after RNase A treatment. However, the amount of nuclear DNA and RNA retained is reduced significantly after RNase A treatment. **Supplementary Figure 2.** Enrichment of MARs after stabilization. The plot shows that the 1321 MARs common between unstabilized and stabilized preparation, are enriched after stabilization. The fold enrichment of stabilized MARs is calculated with respect to unstabilzed MARs. **Supplementary Figure 3.** Analysis of MARs after RNase A treatment. The box plot shows that size of MARs lost after RNAse A treatment is smaller than the one retained or gained. **Supplementary Figure 4.**Majority MARs are histone free regions. Plot shows overlap of H3 ChIP data with MARs prepared by variation in protocol and from different developmental stages. Almost 40-70% of the MARs have no H3 overlap indicating absence of histones over these regions. **Supplementary Figure 5.** Original, uncropped Gels and blots used in Figure 5. PCR amplified MAR DNA sequences were resolved on 1% agarose gel, stained with EtBr and imaged. Panels 1 and 2 were run on upper and lower section of the same gel tray. Similarly 3 and 4 were run on the same gel tray. Panel 5 was run alone. The gels were transferred to nylon membrane, cut into independent panels and hybridized to respective 32P -labeled probe. Probed blots were exposed to Phosphor imaging screen for 12 hours and images captured.

**Additional file 2. Supplementary Table 1. **List of proteins identified by LC-MS/MS of Unstabilized NuMat. Comparison of proteins Stabilized and Unstabilized NuMat.

**Additional file 3: Supplementary Table 2. **Genomic distribution of MARs prepared by variation in protocol. MARs were mapped to euchromatin and pericentromeric heterochromatin. The percent figure has been rounded off to nearest whole number.

**Additional file 4. Supplementary Table 3. **Stabilized MARs (0-16hr Embryos) : 5407 in number. Unstabilized MARs (0-16hr Embryos) : 3832 in number.

**Additional file 5: Supplementary Table 4. **Mapping of MARs prepared by variation in protocol. MARs were annotated in various categories with respect to gene position. The percent figure has been rounded off to nearest whole number.

**Additional file 6. Supplementary Table 5. **PolII binding status on genes with 5'UTR MARs obtained from Stabilized NuMat (0-16 hr embryos) (Zeitlinger et al, 2007, Nat. Genet. 39, 1512 16).

**Additional file 7. Supplementary Table 6. **Early MARs (0-2hr Embryos): 1228 in number. Late MARs (14-16hr Embryos) : 6382 in number.

**Additional file 8: Supplementary Table 7. **Mapping of MARs at different developmental stages. MARs were annotated in various categories with respect to gene position. The percent figure has been rounded off to nearest whole number.

**Additional file 9: Supplementary Table 8. **Protein factors binding at MARs from 0-16 hour embryos. ChIP-Seq data for protein binding has been obtained from modENCODE. Transcription factors (TFs) – lola, Trithorax-like, fruitless, kruppel, knirps, caudal, hairy, homothorax, pangolin, paired, ultraspiracle, Polycomb-like, yorkie, scute, huckebein, Su(H), Hr46, Hr78, Eip74EF, Stat92E, MBD-R2. Insulator binding proteins (IBPs) –BEAF32, CP190, CTCF, Su(Hw), Mod(mdg)4, Zw5. Heterochromatin protein (HPs) – HP1a, HP1b, HP1c, HP2, HP4, Su(var)3-7.

**Additional file 10. Supplementary Table 9. **Core-MARs (0-16hr Embryos) : 1277 in number.

**Additional file 11. Supplementary Table 10. **Details of modENCODE data used for analysis.

**Additional file 12: Supplementary Table 11. **List of primers used for generating PCR products for Southern hybridizations.

## Data Availability

All data generated or analyzed in this study are included in this article and its supplementary information files. The sequencing datasets supporting the conclusion of this article are available in the NCBI Sequence Read Archive (https://www.ncbi.nlm.nih.gov/sra) under accession number PRJNA646046. The mass spectrometry proteomics data is available in the ProteomeXchange Consortium via the PRIDE partner repository (https://www.ebi.ac.uk/pride) with the dataset identifier PXD031279". The data can be accessed using the Username: reviewer_pxd031279@ebi.ac.uk and Password: WhGisbiV.
