# Supplementary material for: Structural and developmental dynamics of Matrix associated regions in Drosophila melanogaster genome
Source: BMC Genomics. 2022 Oct 25;23:725. doi: 10.1186/s12864-022-08944-4 (PMC9597980; doi:10.1186/s12864-022-08944-4)
Supplement: Supplementary file 1 — Additional file 1: Supplementary Figure 1. Molecular composition of NuMat after stabilization and RNase A treatment. Amount of nuclear DNA, RNA and proteins retained in unstabilized, stabilized and RNase A treated NuMat: The amount of nuclear DNA, RNA and proteins retained in unstablized NuMat is significantly less than stabilized NuMat. The amounts of nuclear proteins retained in NuMat does not change after RNase A treatment. However, the amount of nuclear DNA and RNA retained is reduced significantly after RNase A treatment. Supplementary Figure 2. Enrichment of MARs after stabilization. The plot shows that the 1321 MARs common between unstabilized and stabilized preparation, are enriched after stabilization. The fold enrichment of stabilized MARs is calculated with respect to unstabilzed MARs. Supplementary Figure 3. Analysis of MARs after RNase A treatment. The box plot shows that size of MARs lost after RNAse A treatment is smaller than the one retained or gained. Supplementary Figure 4.Majority MARs are histone free regions. Plot shows overlap of H3 ChIP data with MARs prepared by variation in protocol and from different developmental stages. Almost 40-70% of the MARs have no H3 overlap indicating absence of histones over these regions. Supplementary Figure 5. Original, uncropped Gels and blots used in Figure 5. PCR amplified MAR DNA sequences were resolved on 1% agarose gel, stained with EtBr and imaged. Panels 1 and 2 were run on upper and lower section of the same gel tray. Similarly 3 and 4 were run on the same gel tray. Panel 5 was run alone. The gels were transferred to nylon membrane, cut into independent panels and hybridized to respective 32P -labeled probe. Probed blots were exposed to Phosphor imaging screen for 12 hours and images captured. [file 12864_2022_8944_MOESM1_ESM.pdf]

Supplementary Figure 1

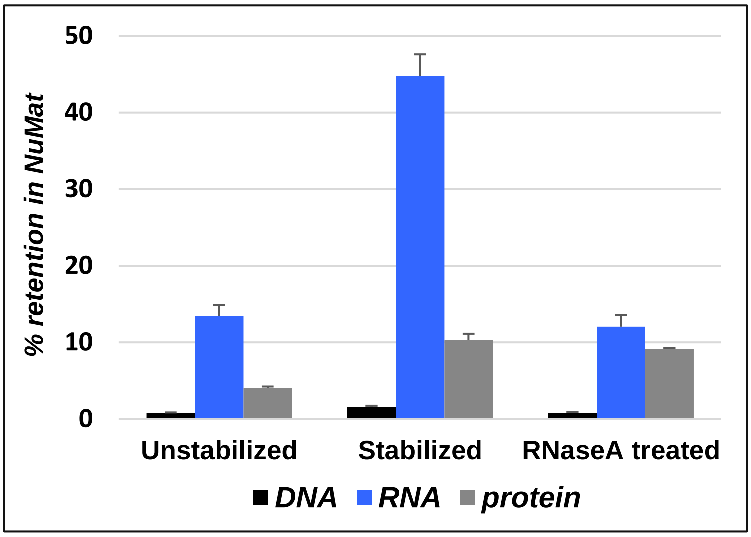

Molecular composition of NuMat after stabilization and RNase A treatment. Amount of nuclear DNA, RNA and proteins retained in unstabilized, stabilized and RNase A treated NuMat: The amount of nuclear DNA, RNA and proteins retained in unstablized NuMat is significantly less than stabilized NuMat. The amounts of nuclear proteins retained in NuMat does not change after RNase A treatment. However, the amount of nuclear DNA and RNA retained is reduced significantly after RNase A treatment.

Supplementary Figure 2

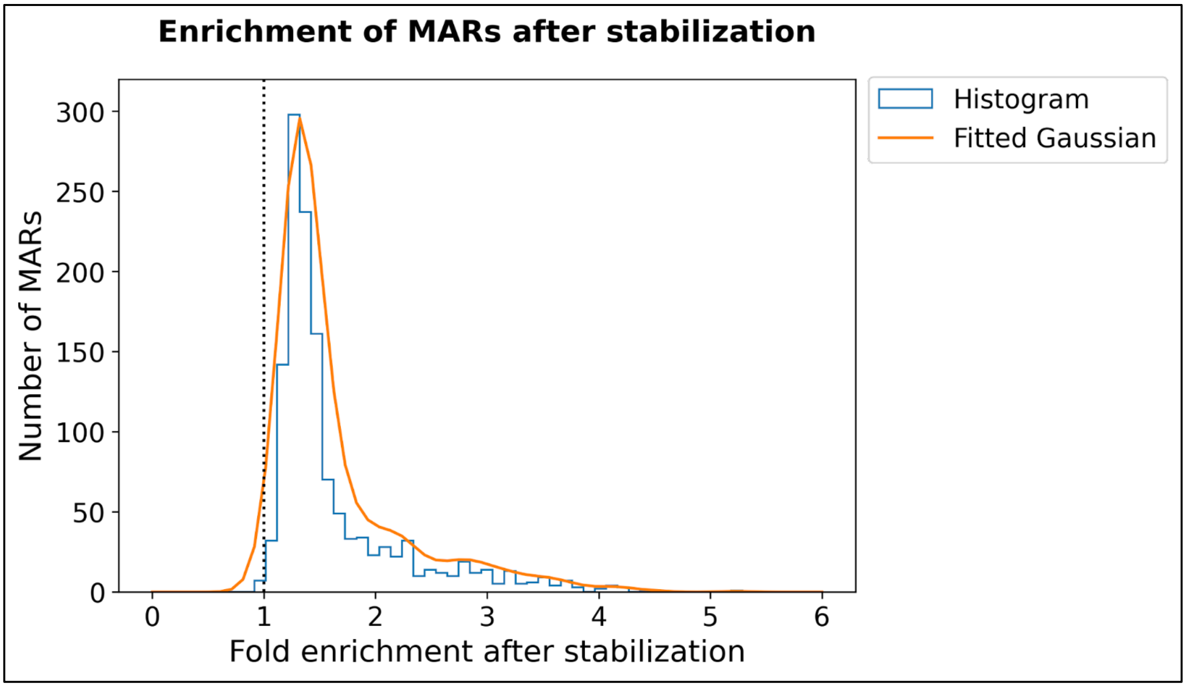

Enrichment of MARs after stabilization. The plot shows that the 1321 MARs common between unstabilized and stabilized preparation, are enriched after stabilization. The fold enrichment of stabilized MARs is calculated with respect to unstabilzed MARs.

Supplementary Figure 3

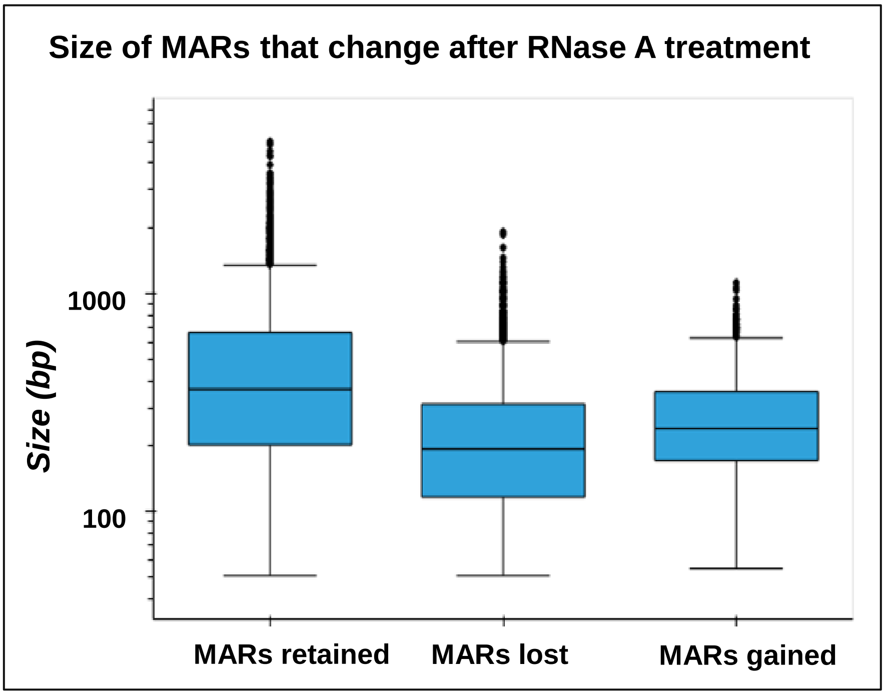

Analysis of MARs after RNase A treatment. The box plot shows that size of MARs lost after RNAse A treatment is smaller than the one retained or gained.

Supplementary Figure 4

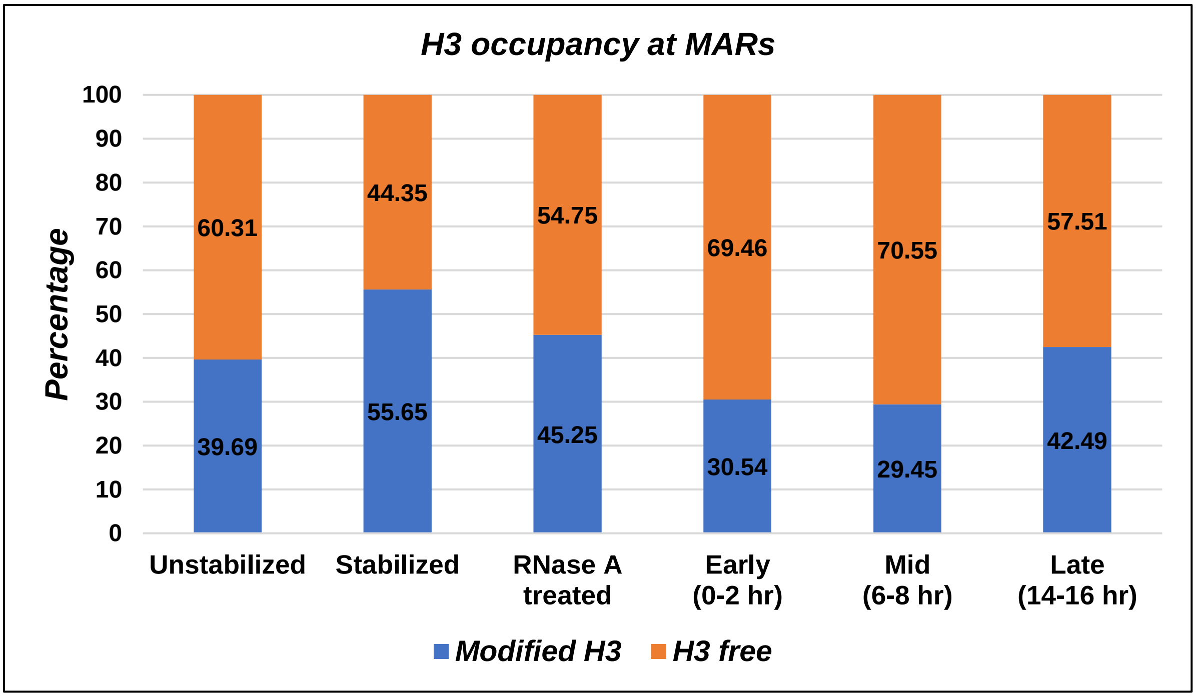

Majority MARs are histone free regions. Plot shows overlap of H3 ChIP data with MARs prepared by variation in protocol and from different developmental stages. Almost 40-70% of the MARs have no H3 overlap indicating absence of histones over these regions.

Supplementary Figure 5

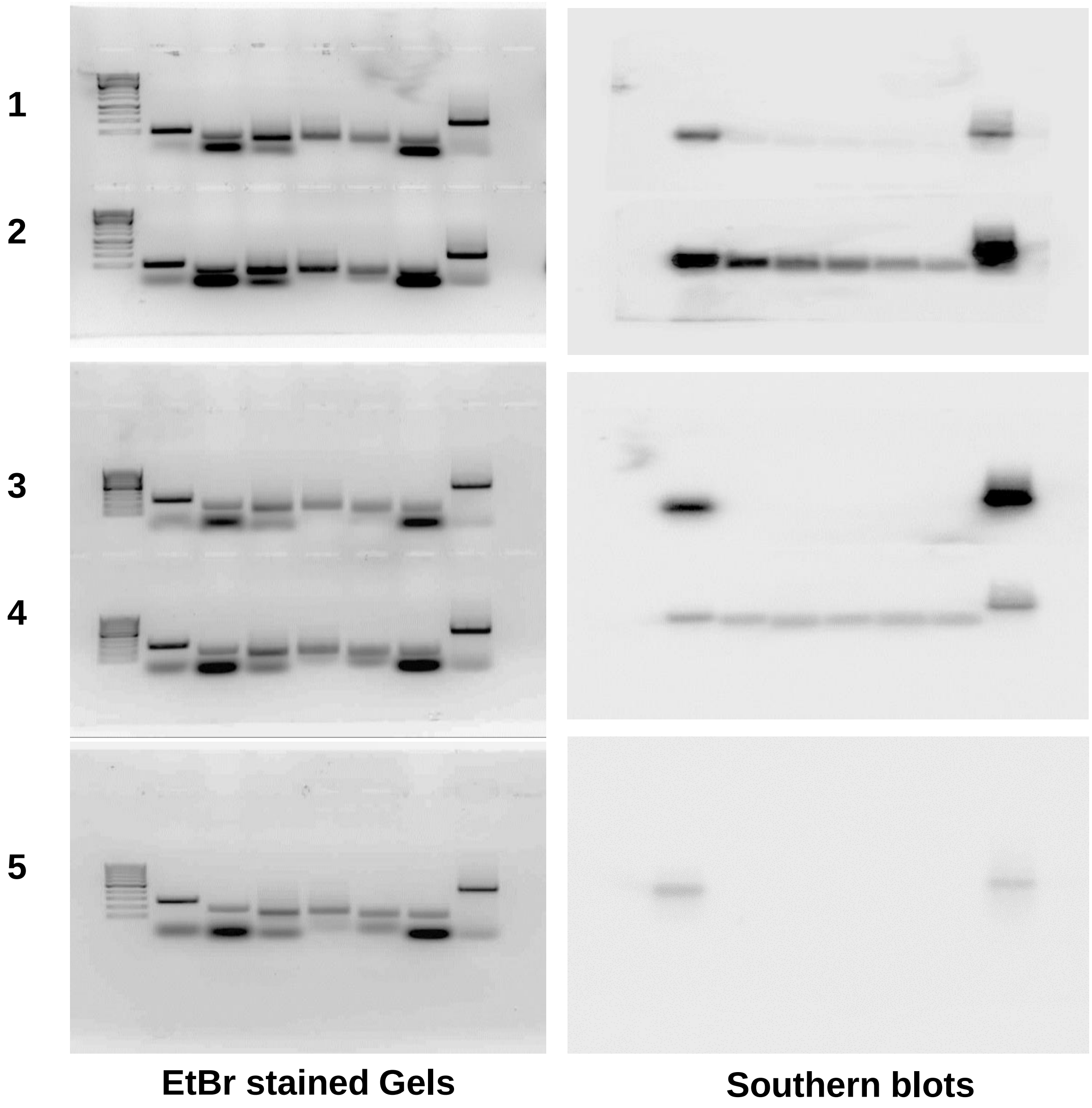

Original, uncropped Gels and blots used in Figure 5

PCR amplified MAR DNA sequences were resolved on 1% agarose gel, stained with EtBr and imaged. Panels 1 and 2 were run on upper and lower section of the same gel tray. Similarly 3 and 4 were run on the same gel tray. Panel 5 was run alone. The gels were transferred to nylon membrane, cut into independent panels and hybridized to respective 32P -labeled probe. Probed blots were exposed to Phosphor imaging screen for 12 hours and images captured.
