# Supplementary material for: Structural and developmental dynamics of Matrix associated regions in Drosophila melanogaster genome
Source: BMC Genomics. 2022 Oct 25;23:725. doi: 10.1186/s12864-022-08944-4 (PMC9597980; doi:10.1186/s12864-022-08944-4)
Supplement: Supplementary file 3 — Additional file 3: Supplementary Table 2. Genomic distribution of MARs prepared by variation in protocol. MARs were mapped to euchromatin and pericentromeric heterochromatin. The percent figure has been rounded off to nearest whole number. [file 12864_2022_8944_MOESM3_ESM.docx]

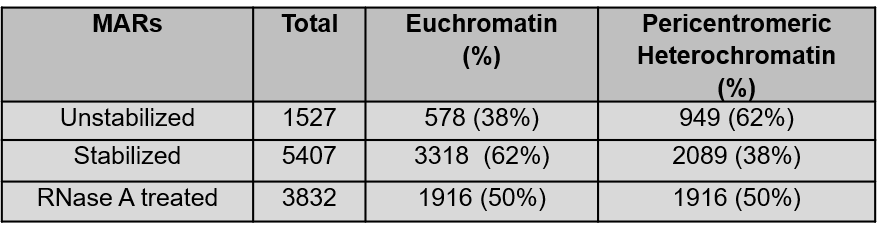


**Supplementary Table 2**

**Genomic distribution of MARs prepared by variation in protocol. MARs were mapped to euchromatin and pericentromeric heterochromatin. The percent figure has been rounded off to nearest whole number.**
