# Supplementary material for: Structural and developmental dynamics of Matrix associated regions in Drosophila melanogaster genome
Source: BMC Genomics. 2022 Oct 25;23:725. doi: 10.1186/s12864-022-08944-4 (PMC9597980; doi:10.1186/s12864-022-08944-4)
Supplement: Supplementary file 5 — Additional file 5: Supplementary Table 4. Mapping of MARs prepared by variation in protocol. MARs were annotated in various categories with respect to gene position. The percent figure has been rounded off to nearest whole number. [file 12864_2022_8944_MOESM5_ESM.docx]

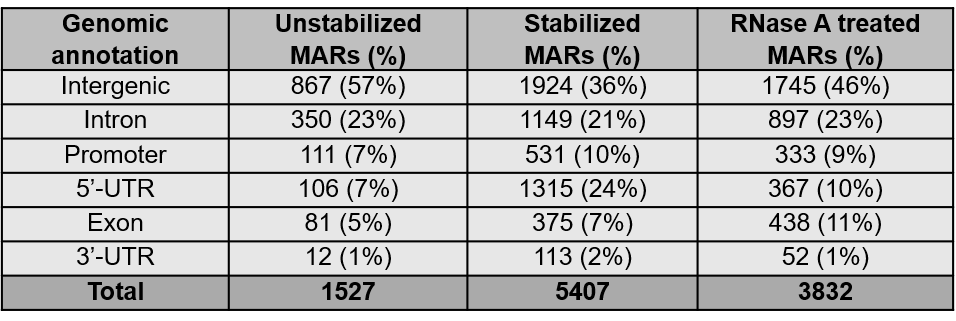


**Supplementary Table 4**

**Mapping of MARs prepared by variation in protocol. MARs were annotated in various categories with respect to gene position. The percent figure has been rounded off to nearest whole number.**
