# Supplementary material for: Structural and developmental dynamics of Matrix associated regions in Drosophila melanogaster genome
Source: BMC Genomics. 2022 Oct 25;23:725. doi: 10.1186/s12864-022-08944-4 (PMC9597980; doi:10.1186/s12864-022-08944-4)
Supplement: Supplementary file 9 — Additional file 9: Supplementary Table 8. Protein factors binding at MARs from 0-16 hour embryos. ChIP-Seq data for protein binding has been obtained from modENCODE. Transcription factors (TFs) – lola, Trithorax-like, fruitless, kruppel, knirps, caudal, hairy, homothorax, pangolin, paired, ultraspiracle, Polycomb-like, yorkie, scute, huckebein, Su(H), Hr46, Hr78, Eip74EF, Stat92E, MBD-R2. Insulator binding proteins (IBPs) –BEAF32, CP190, CTCF, Su(Hw), Mod(mdg)4, Zw5. Heterochromatin protein (HPs) – HP1a, HP1b, HP1c, HP2, HP4, Su(var)3-7. [file 12864_2022_8944_MOESM9_ESM.docx]

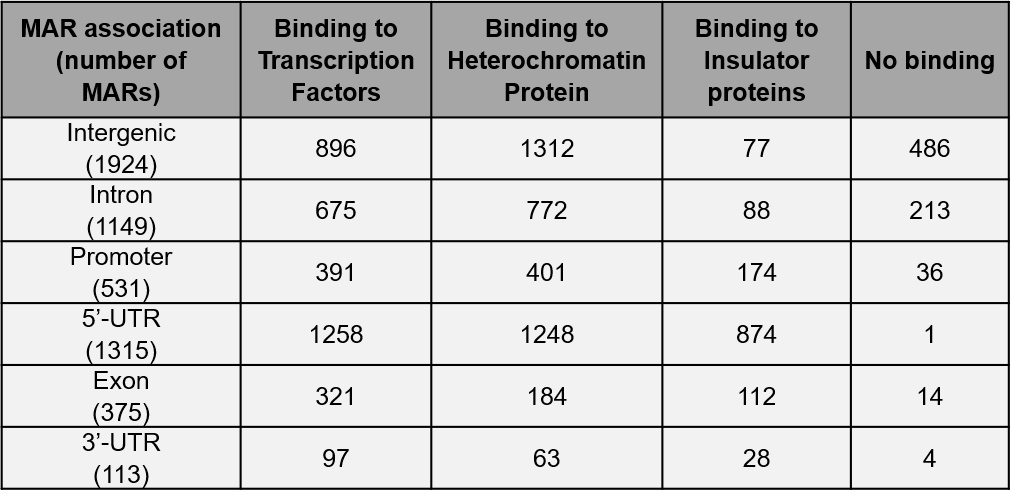


**Supplementary Table 8**

**Protein factors binding at MARs from 0-16 hour embryos.**

***ChIP-Seq data for protein binding has been obtained from modENCODE***

***Transcription factors (TFs) – lola, Trithorax-like, fruitless, kruppel, knirps, caudal, hairy, homothorax, pangolin, paired, ultraspiracle, Polycomb-like, yorkie, scute, huckebein, Su(H), Hr46, Hr78, Eip74EF, Stat92E, MBD-R2***

***Insulator binding proteins (IBPs) – BEAF32, CP190, CTCF, Su(Hw), Mod(mdg)4, Zw5***

***Heterochromatin proteins (HPs) – HP1a, HP1b, HP1c, HP2, HP4, Su(var)3-7***
