# Supplementary material for: Structural and developmental dynamics of Matrix associated regions in Drosophila melanogaster genome
Source: BMC Genomics. 2022 Oct 25;23:725. doi: 10.1186/s12864-022-08944-4 (PMC9597980; doi:10.1186/s12864-022-08944-4)
Supplement: Supplementary file 12 — Additional file 12: Supplementary Table 11. List of primers used for generating PCR products for Southern hybridizations. [file 12864_2022_8944_MOESM12_ESM.docx]

Supplementary Table 11 - List of primers used for generating PCR products for Southern hybridizations

| Primer (F/R) | Sequence |
| --- | --- |
| U1F | ACCTGCTCCAGTTTCTT |
| U1R | ACACGCGGCATACGGAA |
| U2F | AAGAACGTCGTGTGCGTG |
| U2R | GCATCATTCTTTCTCTGC |
| U3F | ATTATCGTATGTGTCGAG |
| U3R | CGATTGGGTTCGATCTCG |
| U4F | CGACATAACCAGCGCCAA |
| U4R | ATAAGTTGTGGGAAATTC |
| U5F | GATTCCCGACGATTTTCT |
| U5R | TGAAATTCGCCTCGCACG |
| C1F | CTGATTTAGCTTGGGAAC |
| C1R | GCGGACTGAACTGTGGAA |
| C2F | GCCCAACTGACACATAA |
| C2R | ATCACGTCCAGTATCTCG |
